# Overcoming the Cost of Positive Autoregulation by Accelerating the Response with a Coupled Negative Feedback

**DOI:** 10.1016/j.celrep.2018.08.023

**Published:** 2018-09-11

**Authors:** Rong Gao, Ann M. Stock

**Affiliations:** 1 Center for Advanced Biotechnology and Medicine, Department of Biochemistry and Molecular Biology, Rutgers University-Robert Wood Johnson Medical School, Piscataway, NJ 08854, USA; 2 Lead Contact

## Abstract

A fundamental trade-off between rapid response and optimal expression of genes below cytotoxic levels exists for many signaling circuits, particularly for positively autoregulated systems with an inherent response delay. Here, we describe a regulatory scheme in the *E. coli* PhoB-PhoR two-component system, which overcomes the cost of positive feedback and achieves both fast and optimal steadystate response for maximal fitness across different environments. Quantitation of the cellular activities enables accurate modeling of the response dynamics to describe how requirements for optimal protein concentrations place limits on response speed. An observed fast response that exceeds the limit led to the prediction and discovery of a coupled negative autoregulation, which allows fast gene expression without increasing steady-state levels. We demonstrate the fitness advantages for the coupled feedbacks in both dynamic and stable environments. Such regulatory schemes offer great flexibility for accurate control of gene expression levels and dynamics upon environmental changes.

## In Brief

Positive autoregulation of transcription produces a delayed response. Gao and Stock describe the limit of response delay caused by requirements of optimal protein levels in the PhoBR twocomponent system. Coupled negative autoregulation is discovered to allow a strong promoter for fast response without incurring cost of increasing protein expression levels.

## Graphical Abstract

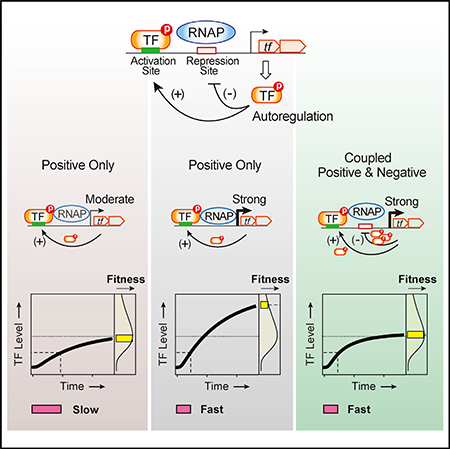

## INTRODUCTION

Cells have evolved complex gene regulatory networks to produce appropriate amounts of proteins at appropriate times to adapt to ever-changing environments. A few recurring network motifs, such as feed-forward loops and autoregulatory circuits, constitute the basic building blocks for more sophisticated regulatory networks (Alon, 2007; Lim et al., 2013; Wall et al., 2004). Molecular mechanisms, dynamic behaviors, and functional roles of these motifs have been extensively studied both experimentally and theoretically (Maeda and Sano, 2006; Mitrophanov and Groisman, 2008; Rosenfeld et al., 2002; Shen-Orr et al., 2002), often in the context of how specific motifs perform certain functions to benefit cells. Less experimental investigation has focused on how biochemical properties place constraints on individual motifs and how cells are evolved to overcome such restrictions. We used a bacterial two-component system to examine how cells balance the benefit and cost of positive autoregulation, a common motif widely distributed in diverse regulatory networks.

Positive autoregulation occurs when a transcription factor (TF) activates its own expression. It has been established that positive autoregulation can increase the sensitivity to signals, produce a switch-like response, and promote bistability, history-dependent hysteretic responses, or memory (Alon, 2007; Mitrophanov and Groisman, 2008; Tiwari et al., 2011; Xiong and Ferrell, 2003). Positive autoregulation or auto-activation of a TF leads to more TF molecules and the consequent amplification of the TF-regulated output response (Mitrophanov et al., 2010; Miyashiro and Goulian, 2008), as well as amplification of noise or cell-cell variations (Chalancon et al., 2012; Miyashiro and Goulian, 2008). All these features could potentially benefit or impair specific pathways. Positive autoregulation also has a significant impact on response dynamics (Maeda and Sano, 2006; Mitrophanov et al., 2010). It has long been predicted, and later experimentally shown, that positive feedback slows down the kinetics of response protein synthesis (Maeda and Sano, 2006; Savageau, 1974) because of the time required to produce the TF to a level sufficient for activation. A slow response may not be desirable for many signaling tasks. In the early days, when examples of auto-activated TFs were still scarce, response speed was suggested as one criterion that selects against positive autoregulation (Savageau, 1974). Since then, many more TFs have been discovered to positively regulate their own expression. Among ~200 characterized TFs in *Escherichia coli*, approximately half are autoregulated, and of these, ~30 TFs are positively autoregulated (Gama-Castro et al., 2016; Hermsen et al., 2010). The frequent occurrence of positive autoregulation suggests that the cost in response speed can be overcome or tolerated. Evolution of these positive autoregulated pathways depends on the cost and benefit defined by diverse response features.

At least 10 of the 30 auto-activated TFs in *E. coli* belong to the family of two-component systems (TCSs) and positive autoregulation is more common than negative autoregulation in TCSs (Goulian, 2010). The TCS is one of the major prokaryotic signal transduction schemes (Bhate et al., 2015; Gao and Stock, 2009). It involves a sensor histidine kinase (HK) that responds to environmental cues and regulates the phosphorylation level of the cognate response regulator (RR), usually a transcription regulator, to adjust output response. Bimodal response at a defined stimulus level or strong history-dependent hysteresis is not commonly observed in TCSs (Groisman, 2016; Tiwari et al., 2011) and presumably not advantageous in most TCS signaling pathways, which may prefer explicit input-output relations. On the other hand, mathematic modeling of TCSs predicts that response speed can be severely slowed by positive autoregulation, presenting a considerable cost (Hermsen et al., 2011). Despite the emerging studies on temporal dynamics of TCSs (Gao and Stock, 2017; Salazar and Laub, 2015; Salazar et al., 2016; Yeo et al., 2012), effects of positive autoregulation on TCS response kinetics have not been well characterized. It is unknown how much response delay is caused by positive autoregulation and whether any mechanism exists to expedite the response.

**Figure 1.**
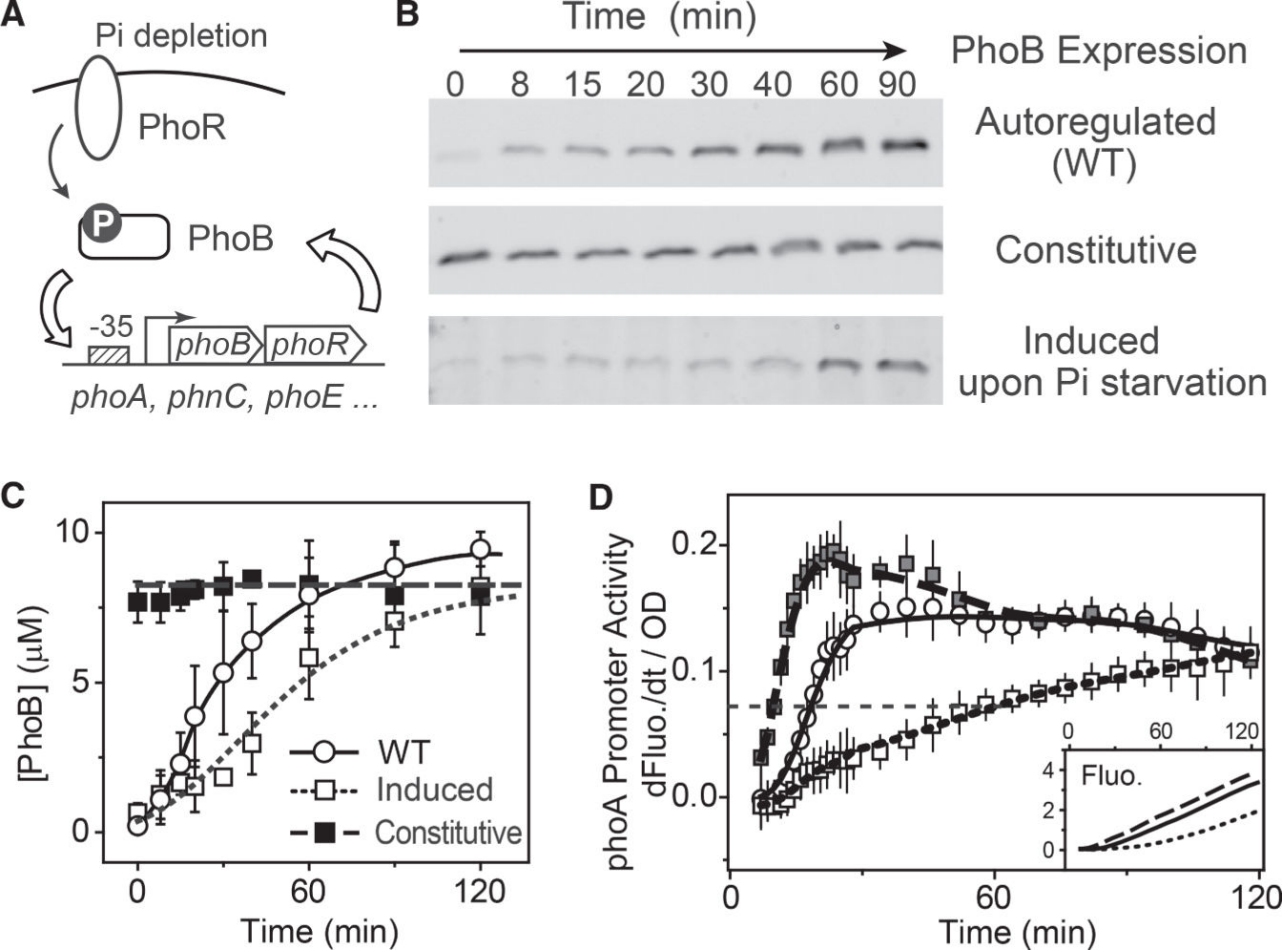
Dependence of Response Kinetics on PhoB Accumulation Rate (A) Schematic diagram of PhoBR autoregulation. (B and C) Time-dependent PhoB expression examined with immunoblots. At time = 0, cells were starved for Pi by resuspension in MOPS medium (2 μM Pi). For comparison with the expression kinetics of the autoregulated WT (BW25113), PhoB levels of RU1616 were constantly maintained with 150 μM IPTG (constitutive) or induced by adding 150 μM IPTG at the start of Pi starvation (induced). One representative of at least two immunoblots is shown in (B). Data in (C) are shown as mean ± SD from quantifications of at least two immunoblots. (D) Response kinetics of Pi starvation examined using the *phoA-yf*p reporter plasmid pRG161. Fluorescence traces are shown in the inset. OD-normalized first derivatives of fluorescence are used to represent promoter activity. Data are shown as mean ± SD from 11 individual wells; symbols are as indicated in (C).

We studied the activation dynamics of the archetype PhoB/ PhoR system in *E. coli*. The sensor HK PhoR responds to limitation of environmental phosphate (Pi) concentrations and modulates its activities, including the autokinase, phosphotransferase, and phosphatase activities, to control the phosphorylation level of the RR transcription factor PhoB (Wanner, 1996). Phosphorylated PhoB activates expression of the *phoBR* operon, as well as other genes that are responsible for phosphorus assimilation, including *phoA*, encoding an alkaline phosphatase and the *phn* operon encoding phosphonate utilization genes (Figure 1A). Steady-state expression levels of PhoB have strong effects on cell fitness. We have shown previously that Pi-depleted and -replete conditions select for different steady-state PhoB expression levels, which correlate with intrinsic enzyme activities and are optimized for RR phosphorylation (RR~P) output with minimized cost of protein production (Gao and Stock, 2013a). Positive autoregulation of *phoBR* benefits cells by providing the capability to switch between different optimal PhoB levels for maximal fitness across a range of environments.

In this study, by measuring *in vivo* dynamics of PhoB phosphorylation and expression, we were able to develop a quantitative model, evaluate promoter features that affect response speed, and predict the autoregulation kinetics. We show that positive autoregulation in wild-type (WT) cells causes a response delay, however, the delay is not as severe as predicted by the model. A coupled negative feedback through auto-repression of the *phoB* promoter was discovered to overcome the cost of positive autoregulation by enabling promoter features that reduce the response delay. Unlike autoregulatory variants that have only positive feedback and cannot resolve the trade-off between response speed and amplitude, the coupled feedbacks enable WT cells to achieve fast response speed as well as optimal expression levels for greater fitness.

## RESULTS

### Autoregulation of PhoB Causes Only a Slight Delay in Response

To investigate how positive autoregulation affects the response speed of the *E. coli phoBR* system, we examined the activation dynamics of the *phoA-yfp* reporter upon Pi starvation. The *phoA* promoter activity is calculated as the optical density (OD) normalized first derivative of YFP fluorescence and it has been shown to faithfully track with PhoB phosphorylation output (Gao and Stock, 2017). A non-autoregulatory strain, RU1616, was used for comparison because it contains an isopropyl β-D-1-thiogalactopyranoside (IPTG)-inducible *lac* promoter for the *phoBR* operon and can produce a wide range of PhoB and PhoR concentrations (Gao and Stock, 2013b). The PhoR expression level has been shown to be approximately one tenth of the PhoB concentration when both are expressed from the same operon (Gao and Stock, 2013b), thus, we use PhoB levels to probe expression of the entire *phoBR* operon.

When cultures contained 150 μM IPTG throughout the assay, a constant PhoB expression level was maintained at a level comparable to the steady-state PhoB level of WT under Pi-depleted conditions (Figures 1B and 1C), representing constitutive expression of *phoBR*. Not surprisingly, this strain displayed a faster response than WT (Figure 1D) because unlike the autoregulated WT, it requires no time for accumulation of PhoB protein. It appears that the positive autoregulation in WT caused only a slight delay, <10 min in half-time for the rise of promoter activity.

In the absence of IPTG, RU1616 produces a PhoB concentration similar to the basal level of WT under Pi-replete conditions. Addition of 150 μM IPTG at the start of Pi starvation induces PhoB expression along with phosphorylation-mediated activation of the PhoB/PhoR system, and as in autoregulated WT, time is required for PhoB protein to accumulate to the steadystate level. However, the dynamics of *phoA* promoter activation for this induced condition differ greatly from those of WT, exhibiting a significant response delay. It requires longer than 60 min for the promoter activity to rise to 50% of the final level (Figure 1D). The delay is unlikely due to the uptake of IPTG because IPTG or other inducer molecules of the *lac* promoter enter the cell at a much shorter timescale (Elf et al., 2007; Stamatakis and Mantzaris, 2009). This suggests that simultaneous induction of the regulator expression with signal activation, one of the signature traits of positive autoregulation, could elicit considerable costs in response speed in comparison to a constitutively expressed system. The response speed appears to be correlated with the accumulation rate, or the expression kinetics of the transcription regulator PhoB. The WT strain expressed PhoB faster than the induced sample, thus showed less delay in response, even though steady-state PhoB levels were similar between the induced sample and WT at both start (0 min) and end (90 min) of the assay (Figures 1B and 1C). The fast expression kinetics of PhoB may help WT cells reduce the response delay of positive autoregulation and gain fitness advantages. We are interested in the intrinsic mechanism that controls the dynamics of autoregulation and the corresponding fitness cost and benefit of autoregulation.

### PhoB Expression Kinetics Are Faster Than Predicted by the Model

**Figure 2.**
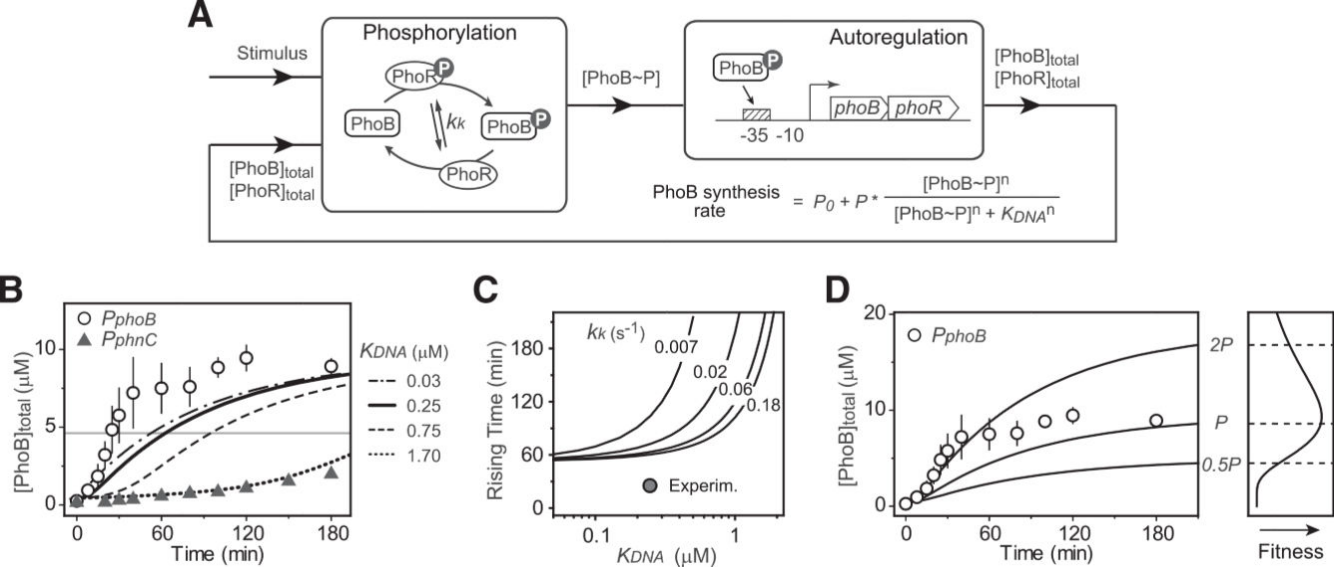
Effects of TF DNA-Binding Affinity and Promoter Strength on PhoB Expression Kinetics (A) Phosphorylation and transcription feedbackmodules for the autoregulation model (see details in Figure S1). (B) Slower PhoB kinetics caused by weaker DNA affinity. PhoB expression kinetics modeled with different affinities are shown in curves. The solid and dotted curves were modeled with the affinities of PhoB~P to the phoB (K_DNA_ = 0.25 mM) and phnC (K_DNA_ = 1.7 μM) promoters. Affinities were measured by EMSAs (Figure S2). The horizontal line indicates the half-maximal PhoB level. Symbols represent PhoB expression kinetics experimentally measured (Figure S3) for RU1878 (*P*_*phoB*_) and RU1881 (*P*_*phnC*_). Data are shown as mean ± SD from five (*P*_*phoB*_) or four (*P*_*phnC*_) experiments. RU1881 is engineered to express *phoBR* from the *phnC* promoter. RU1878 and RU1881 have identical genetic backgrounds. The WT *phoBR* operon in RU1878 is not at its original location; however, RU1878 displayed identical expression kinetics as the WT strain BW25113 (Figure S3). (C) Dependence of rising time on promoter affinity. Rising time was calculated as the time required to reach the half-maximal PhoB level, 4.7 μM. Solid curves indicate the rising times calculated with different values of the autophosphorylation kinetics parameter k_k_. WT has a k_k_ of 0.06 s^−1^. (D) Effects of promoter strength on autoregulation kinetics and fitness. Solid lines represent modeled data with indicated promoter strengths. The value of *P* is set at 20 × *P*_0_. Open circles show the experimental data for RU1878, as in (B). The curve in the fitness sub-panel is simulated with a log-normal curve that illustrates the fitness trend of previous data (Gao and Stock, 2013a).

PhoB expression kinetics are dependent on the autoregulatory transcription of *phoBR*, which is tightly coupled with the phosphorylation reactions and difficult to characterize in isolation. To comprehensively understand the controlling mechanism of autoregulation dynamics, we modeled the system with two interconnected modules, the phosphorylation/dephosphorylation cycle that regulates the output concentration of phosphorylated PhoB (PhoB~P) and the transcription feedback that determines the expression of phoBR (Figure 2A). The PhoB expression rate is described by a Hill equation with the Hill coefficient *n* set at 2 to reflect a single PhoB~P binding site in the promoter and the fact that PhoB~P binds each site as a dimer (Blanco et al., 2012; Ritzefeld et al., 2013). Kinetics of total PhoB concentrations are determined by the degradation/dilution rate (STAR Methods) as well as the PhoB synthesis rate. Multiple parameters, such as the concentration of PhoB~P, the binding affinity of PhoB~P to the promoter and the auto-activated *phoB* promoter strength *P*, are able to adjust the PhoB production rate and thus influence the expression kinetics.

The intrinsic enzyme activities of PhoR and PhoB proteins determine the dynamic change of PhoBP concentrations. The phosphorylation cycle was modeled as described previously (Batchelor and Goulian, 2003; Gao and Stock, 2013b; Siryaporn et al., 2010) considering multiple enzyme reactions including PhoR autophosphorylation, phosphotransfer to PhoB and the dephosphorylation of PhoB~P (Figure S1A). *In vivo* phosphorylation kinetics at three different constant PhoB levels were measured with the non-autoregulatory strain to decouple phosphorylation from *phoBR* expression (Figure S1). Parameter values were estimated to generate phosphorylation kinetics recapitulating the experimental data (see details in STAR Methods and Table S1). With all of the phosphorylation parameters determined, it becomes possible to assess quantitatively how the promoter-specific factors, such as the promoter strength and binding affinity, affect the PhoB autoregulation kinetics.

We first examined the effects of promoter binding affinity on PhoB expression kinetics. The dissociation constant for promoter binding, K_DNA_, was determined by electrophoretic mobility shift assays (EMSAs) (Figure S2). The affinities measured *in vitro* were discovered to be within the same magnitude as those determined by cellular reporter assays (Gao and Stock, 2015) and thus were used for modeling. DNA-binding affinity has a dramatic effect on PhoB expression kinetics (Figures 2B and 2C). Strengthening the promoter binding can speed up the PhoB expression kinetics. Low affinity, i.e., a high value of K_DNA_, can greatly slow down the kinetics. To validate this prediction experimentally, we replaced the WT promoter of *phoBR* with the *phnC* promoter, which has a transcription output comparable to the WT *phoB* promoter and a K_DNA_ value of 1.7 μM, ~7-fold greater than that of the phoB promoter (Figure S2). The resulting operon was incorporated into the chromosome at the HK022 phage attachment site (Haldimann and Wanner, 2001) to generate the autoregulatory variant *P*_*phnC*_. The measured PhoB expression kinetics of this variant were extremely slow, agreeing very well with the modeled data (Figures 2B and S3).

In contrast, a similarly constructed WT allele (*P*_*phoB*_) expressed PhoB rapidly and the kinetics were much faster than the model predicted (Figure 2B). The rising time, defined as the time required for reaching the half-maximal PhoB expression level, is ~25 min, much shorter than the 60 min rising time predicted by the model. The discrepancy between the experimental and modeled data is not due to under-estimation of the binding affinity or the phosphorylation kinetics. Further increase of the affinity (e.g., a decrease of K_DNA_ values from 0.25 μM to 0.03 mM) showed only a marginal increase in PhoB expression speed (Figure 2B). The modeled rising time reaches a limit of ~60 min at low K_DNA_ values (Figure 2C). Increase of phosphorylation kinetics only alters the dependence of rising time on K_DNA_ but does not significantly reduce the rising time limit. As indicated by the Hill equation, when the PhoB expression level increases, PhoB~P concentrations become much larger than the low values of K_DNA_ and the PhoB synthesis rate is close to the maximum. Thus, there is a limit to how much kinetics acceleration can be achieved by a simple increase of promoter affinity or phosphorylation kinetics. It appears that PhoB expression kinetics in WT exceed this limit, and an additional mechanism may mediate the fast response.

The maximal expression speed of PhoB is correlated with the promoter strength *P* (i.e., the maximum transcription rate for the autoregulatory promoter). Autoregulated promoter strength determines both the kinetics and steady-state expression level of PhoB. High promoter strength can increase the expression speed of PhoB and reduce the rising time (Figure 2D). Modeling was performed with parameter values for basal and activated promoter strength chosen based on the steady-state expression levels of PhoB (see details in STAR Methods and Table S1). Doubling the promoter strength *P* leads to matching of modeled PhoB expression kinetics with the experimental data for the initial period (~30 min) of Pi starvation, but it eventually results in a higher steady-state PhoB level than that of WT (Figure 2D). Increased PhoB expression carries a fitness cost. We have shown previously that PhoB expression much higher than the WT level lowers cell fitness (Gao and Stock, 2013a). To reduce the fitness cost of PhoB overproduction and maintain an optimal concentration of PhoB, one possible solution is for WT cells to repress PhoB expression at late stage of Pi starvation. This potential repression would allow WT to have high promoter strength to speed up the response without incurring the cost of high protein expression.

### *phoB* Promoter Is Repressed by PhoB Itself at High Expression Levels

**Figure 3.**
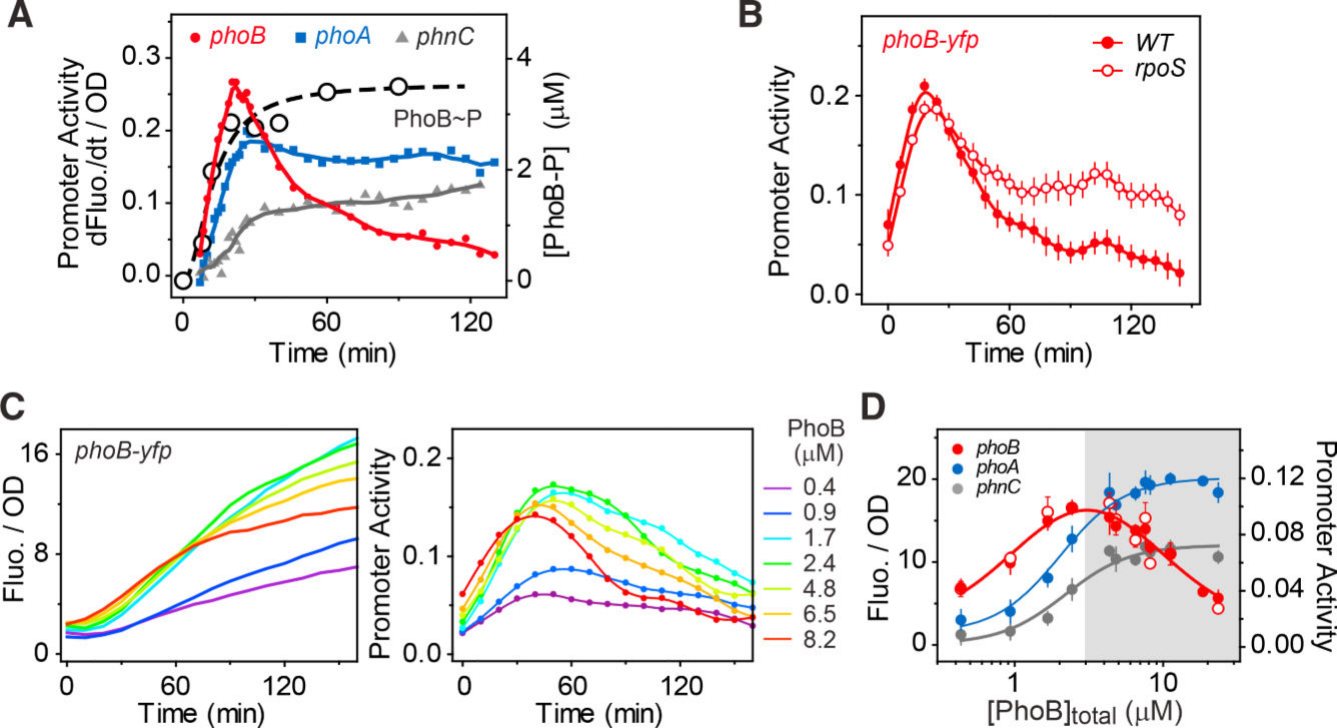
Auto-repression of the *phoB* Promoter (A) Repression of phoB promoter activity during the late stage of Pi starvation. Net promoter activities of *phoB-yfp* (pJZG202), *phoA-yfp* (pRG161), and *phnC-yfp* (pRG162) were assayed in the WT strain BW25113 and calculated as the first derivatives of fluorescence. Solid symbols show the average of 11 individual wells and solid lines illustrate the smoothed data. Open circles with a dashed line show the phosphorylation kinetics measured previously (Gao et al., 2017). (B) Repression of *phoB-yfp* in the *rpoS* deletion strain RU1646. Promoter activity data are shown as mean ± SD from 22 individual wells. (C) Dependence of *phoB* repression on PhoB levels. Reporter dynamics were measured in the non-autoregulatory strains RU1616 or RU1783 at different IPTG concentrations that yield the indicated PhoB levels. Time zero represents the onset time of Pi starvation. One representative sample for each indicated PhoB level is shown. (D) Dependence of reporter output on PhoB expression levels. Promoter activity of *phoB-yfp* (open circles) and OD-normalized fluorescence of three YFP reporters (solid symbols) were measured at 90 min after the onset of starvation and plotted against PhoB concentrations. The shaded area indicates the PhoB concentration range that displayed *phoB* promoter repression. Data are shown as mean ± SD from at least four independent experiments.

To investigate whether the *phoB* promoter is repressed at a late stage of Pi starvation, we measured the activation dynamics of the *phoB-yfp* reporter. The OD-normalized first derivative of fluorescence reflects the promoter activity (i.e., the protein expression rate). Indeed, *phoB* promoter activity decreased sharply after the initial increase upon Pi starvation (Figure 3A). The decrease is not due to dephosphorylation of PhoB~P because PhoB~P showed a monotonic increase throughout Pi starvation. Another two PhoB-regulated promoters, *phoA* and *phnC,* did not show such large decreases in promoter activity, suggesting that the repression is not some global effect on gene expression, but rather specific to the *phoB* promoter. Previous analyses indicate that *phoB* transcription can be inhibited by the stress sigma factor RpoS during the stress response (Gao et al., 2017; Taschner et al., 2004). However, in an *rpoS* deletion strain, significant repression is still present (Figure 3B), albeit to a lower extent than in WT, suggesting an additional inhibitory mechanism for *phoB* expression.

Considering the gradual accumulation of PhoB protein that accompanies system stimulation, we hypothesized that *phoB* expression may be repressed by the PhoB protein itself when the PhoB level becomes high during the late stage of Pi starvation. If this is the case, high constitutive expression of PhoB will lower the reporter output. We examined *phoB-yfp* reporter output in the non-autoregulatory strain at different PhoB expression levels (Figure 3C). At low PhoB levels (e.g., 0.4 or 0.9 μM), promoter activity rises gradually upon Pi starvation and repression is not apparent. Increased PhoB concentrations caused higher initial induction of promoter activity and greater subsequent repression. The higher the PhoB concentration, the earlier the promoter repression occurs and the greater the repression becomes. Such PhoB-dependent repression is not present for the *phoA* and *phnC* promoter (Figures 3D and S4). Once the total PhoB level is above a certain threshold, *phoA* and *phnC* reporter outputs reach saturation and become insensitive to variations of total PhoB concentrations, consistent with the discovery of concentration robustness in TCS phosphorylation (Batchelor and Goulian, 2003; Miyashiro and Goulian, 2008). In contrast, in the non-autoregulatory strain both the promoter activity and the fluorescence output of the *phoB* promoter peaked at an intermediate PhoB concentration while high PhoB expression reduced the output (Figure 3D), agreeing with our hypothesis of auto-repression of *phoB* and suggesting that observations of response robustness can be affected by additional regulation on specific reporter promoters.

**Figure 4.**
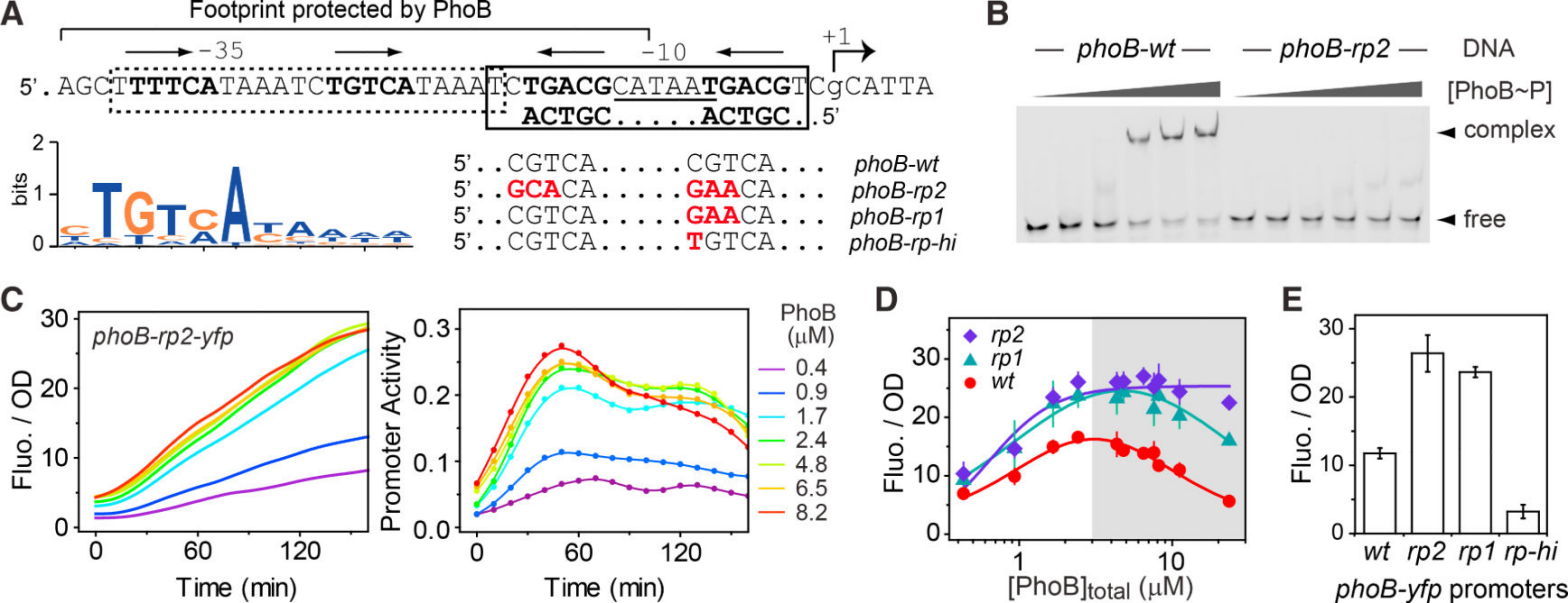
Identification of an Auto-repression Site in the phoB Promoter (A) Illustration of PhoBP binding sites. Sequence of the *phoB* promoter region is shown with the 35, 10, and transcription start (+1) labeled. The dotted box indicates the PhoB activation site, Pho box. Bold letters mark the highly conserved sequence tracts for major groove binding and the arrows above indicate the orientation of the tandem half-sites. The sequence logo of the half-site was generated with the position-weighted matrix that was used for identification of PhoBbinding sites. An additional site (solid box) was identified on the antisense strand and the reverse complementary sequence is shown. Sequence alignments show the major groove sites of WT as well as mutants designed to disrupt (*rp2* and *rp1*) or enhance (*rp-hi*) the binding of PhoB~P to the newly identified site. (B) Binding of PhoB~P to the repression site. EMSA was done using the indicated DNA fragments with PhoB~P concentrations at 0, 1, 2.1, 4.2, 6.3, and 8.4 mM, analyzed in successive lanes from left to right. (C–E) Correlation of *phoB* repression with mutations in the PhoB-binding site. Reporter activities of *phoB-rp2-yfp* (pRG368), *phoB-rp1-yfp* (pRG369), and *phoBrp-hi-yfp*) (pRG367) were examined in the non-autoregulatory strains RU1616 or RU1783 at the indicated PhoB levels. OD-normalized fluorescence and promoter activities of *phoB-rp2-yfp* following the onset of Pi starvation are shown for a representative experiment (C). OD-normalized fluorescence at 90 min after the onset of starvation are shown in (D) across different PhoB levels and in (E) at a concentration of 8.2 mM, a level close to the WT level under Pi-depleted conditions. Data in (D) and (E) are shown as mean ± SD from at least four independent experiments.

We searched the *phoB* promoter sequence for potential PhoB-binding sites that may be responsible for repression (Figures S5A and S5B). Sequences of PhoB-binding sites have been well characterized (Gao and Stock, 2015; Wanner, 1996; Yang et al., 2012). A full site is constituted of two tandem half-sites and each half-site contains a conserved TGTCA tract for major groove binding (Blanco et al., 2002). Two consecutive half-sites overlapping by 1 bp have been identified around the 10 region of the *phoB* promoter (Figure 4A), consistent with previous footprint data that showed a region upstream of 10 was protected by PhoB protein at high concentrations (Makino et al., 1988). EMSA data confirmed the binding of PhoB~P to this site with a weak affinity (K_D_ = 4.9 μM) (Figures 4B and S2). Mutations in the highly conserved positions at both half-sites yielded an oligo (*phoB-rp2*) that displayed little binding to PhoB~P (Figure 4B). The tandem arrangement of half-sites results in a 6-bp spacing between the two TGTCA tracts in a typical PhoB site. However, the newly discovered site has only 5 bp separating the two conserved tracts, which may contribute to its low affinity.

The low affinity of the new site suggests that PhoB~P binds to the 10 region at high concentrations, blocking access of RNA polymerase to the promoter. This is consistent with the observed auto-repression at high PhoB levels. When mutations were made in both half-sites but not the 10 region to disrupt only the PhoBbinding capability, the resulting *phoB-rp2* promoter did not show any significant repression at most PhoB levels (Figure 4C). Promoter activity showed a slow decrease at a late stage in starvation only at the highest PhoB concentration of 8.2 μM. The exact mechanism for this decrease was not explored further but may be attributable to the residual PhoB-binding capacity of *phoB-rp2*. Reporter fluorescence increased along with increased expression levels of PhoB, plateauing at high levels with minimal repression (Figure 4D). Most importantly, the *phoB-rp2* promoter displayed greater reporter fluorescence than WT *phoB,* suggesting relief of repression by the disruptive mutations.

It appears that the extent of repression correlates with the binding capacity of the repression site. Disruption of both halfsites in *phoB-rp2* almost completely abolished the repression. Disruption of only one half-site in *phoB-rp1* also elevated reporter fluorescence but partial repression was still observed at high PhoB levels (Figure 4D). On the other hand, mutating one of the half-sites to the consensus sequence in *phoB-rp-hi* resulted in further inhibition of reporter output (Figure 4E), possibly due to the enhanced binding affinity that strengthens the repression. Taken together, this newly identified PhoB-binding site at the 10 region appears to be responsible for auto-repression of the *phoB* promoter.

### Response Acceleration by Auto-repression Agrees with the Model Prediction

**Figure 5.**
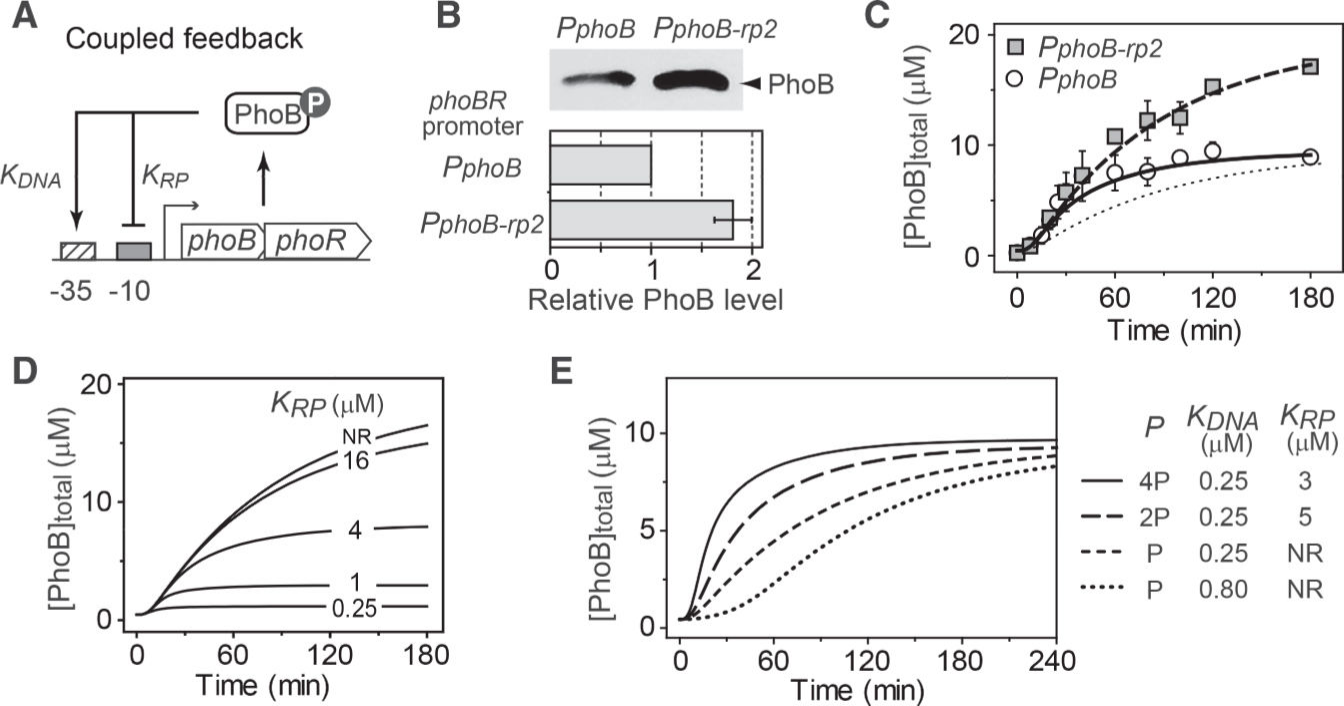
Acceleration of Response Kinetics with Coupled Negative Feedback (A) Schematic diagram of the coupled feedback with both activation and repression sites in the same promoter. (B) Increased PhoB expression in the repression mutant. PhoB expression levels 3 hr after the onset of starvation were quantitated by immunoblotting for the repression mutant strain RU1879 (*PphoB-rp2*) and the corresponding WT strain RU1878 (*PphoB*). Average and SD from eight experiments are shown. (C) Recapitulation of PhoB autoregulation kinetics with the coupled feedback model. Experimentally measured PhoB expression kinetics for the WT and mutant autoregulatory strains are shown in circles and squares as average and SD from at least three experiments (details in Figure S3). The dotted line indicates kinetics that are modeled with the promoter strength parameter, *P*, valued at 20 × *P0* without any repression. The other two lines are modeled with *P* valued at 41 × *P0* without (dashed line) or with (solid line) the coupled negative feedback. (D) Relationship of PhoB expression to the affinity of PhoB for the repression site. All curves are modeled with the value of KDNA at 0.25 μM and the value of *P* at 41 × *P0*. (E) Adjustment of the autoregulation kinetics with the coupled feedback. Parameters for the modeled curves are selected to produce comparable steady-state PhoB expression levels. NR, no repression.

Given the presence of a repression site in addition to the previously established activation site, a coupled feedback model was built to understand expression kinetics of the *phoBR* operon (Figure 5A). Once the system is activated by Pi starvation, positive feedback dominates initially at low PhoB~P concentrations because of the high affinity (K_DNA_) of PhoB~P for the activation site. Low affinity for the repression site (K_RP_) causes the negative feedback to be effective later when PhoB~P becomes sufficiently abundant to occupy the 10 region.

Kinetics modeling of this coupled feedback system requires estimation of the unrepressed promoter strength. At a level of PhoB comparable to that during Pi-depletion, reporter fluorescence of the repression mutant promoter, *phoB-rp2*, is ~2-fold that of WT (Figure 4E). In agreement, under Pi-depleted conditions, *P*_*phoB-rp2*_, the autoregulatory variant with the *phoB-rp2* promoter, expressed PhoB protein at ~2-fold higher levels than WT (Figures 5B and S3). Thus, promoter strength *P* was set at approximately two times the original value. In the absence of repression, the modeled PhoB expression kinetics matched very well with the experimentally measured kinetics of the repression mutant, *P*_*phoB-rp2*_ (Figure 5C). The negative feedback is modeled by limiting the Pho~B synthesis rate with an inhibitory Hill function that reflects the binding of PhoB~P to the repression site. Incorporation of this repression function lowers the steady-state PhoB expression level but maintains the same promoter strength as *P*_*phoB-rp2*_, enabling WT to express PhoB as fast as *P*_*phoB-rp2*_. The modeled kinetics of WT agrees well with the experimental expression kinetics (Figures 5C and S3E). The coupled negative feedback allows high promoter strength to support fast expression kinetics without the cost of protein overproduction.

The modeled PhoB production rate is reduced by auto-repression after the initial increase (Figure S3F) but the extent of reduction is not as great as that shown by the *phoB-yfp* promoter activity (Figure 3A). The observed greater inhibitory effect is likely due to the RpoS-mediated stress response that is not considered in the model. When the stress response is modeled as a general inhibition of transcription, a further reduction of PhoB production rate is apparent (Figure S3F) while the overall PhoB expression levels do not deviate significantly from the experimental data (Figure S3E). Stringent responses induced by nutrient limitation typically exert sophisticated control on different promoters, thus more experimental measurements are required to correctly model the response and for extension to other promoters. Nevertheless, our current model indicates that the complex autoregulatory behavior of WT can be quantitatively understood with the coupled feedbacks.

Auto-repression of the *phoB* promoter provides another mechanism to adjust the steady-state expression level of PhoB. For a given promoter strength, raising the PhoB binding affinity for the repression site reduces PhoB expression (Figure 5D). A relatively weak affinity of K_RP_ = 4 μM is sufficient to give more than 50% repression. High affinity (e.g., a dissociation constant of 0.25 μM that is equal to the affinity for the activation site) leads to very low expression of PhoB. This coupled feedback system offers multiple adjustable elements that provide great flexibility in controlling expression kinetics and output levels. As discussed earlier, an optimal PhoB expression level requires a corresponding promoter strength, which places a limit on PhoB expression rising time in a model with only positive feedback. The coupled negative feedback allows high promoter strength to exceed the rising-time limit. Combinations of different values of promoter strengths and PhoB affinities can enable a full range of diverse expression kinetics, all with similar steady-state PhoB levels (Figure 5E), demonstrating the versatility of the coupled feedback system.

### Cells with Coupled Feedbacks Have High Fitness

For a system with only positive feedback, there is a trade-off between the steady-state expression level and response speed. High promoter strength gives fast response kinetics but results in PhoB levels higher than the optimal concentration. Lowering the promoter strength satisfies the optimal concentration requirement but carries significant cost in response delay. The coupled negative feedback allows WT cells to have the optimal PhoB expression level as well as fast response kinetics. We examined whether the negative feedback gives WT cells fitness advantages over the corresponding autoregulatory variants with only a positive feedback.

**Figure 6.**
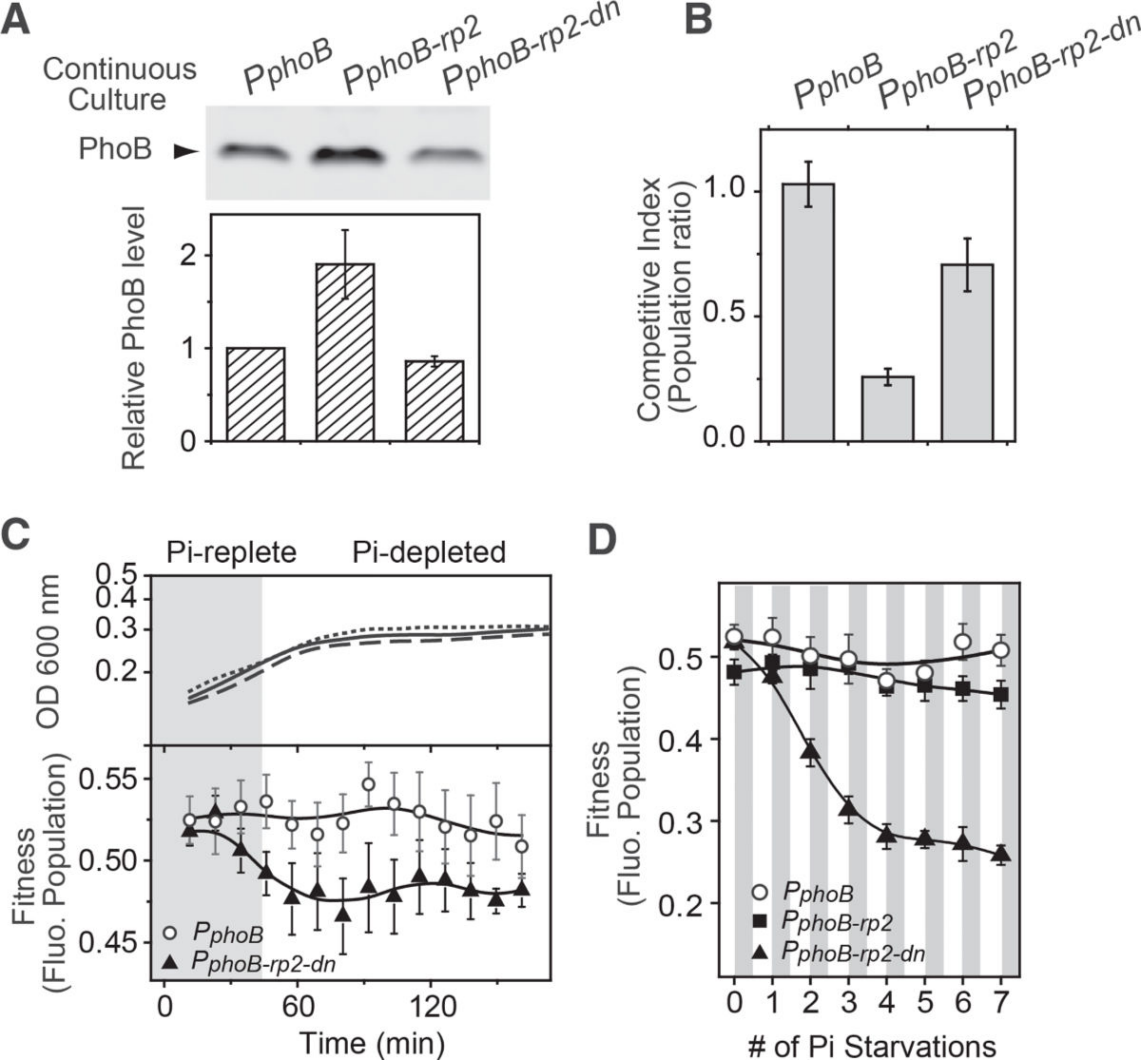
Superior Fitness of the WT Strain with Coupled Feedback (A and B) Steady-state expression levels of PhoB (A) and fitness (B) of autoregulatory variants in Pi-depleted continuous cultures. Levels of PhoB in RU1878 (*P*_*phoB*_), RU1879 (*P*_*phoB-rp2*_), and the RU1879-derivative, RU1880 (*P*_*phoB-rp2-dn*_, see details in Figure S5) were quantitated by immunoblotting. Levels of PhoB in the mutant strains were determined relative to that in RU1878 and data are shown as mean ± SD from four independent experiments. The three above strains carrying a CFP-expressing plasmid pRG411 were competed against RU1878 carrying pRG426, which expresses a non-fluorescent CFP variant. Fluorescent bacterial populations were quantified using a thresholding algorithm (Figure S6). Fitness was calculated as the ratio of the fluorescent bacterial population after competition over the population before competition. (C and D) Bacterial competition in batch cultures. Indicated strains carrying a CFP-expressing plasmid pRG411 were competed against RU1878/pRG426 in batch cultures with a starting Pi concentration at 50 μM. Shaded areas illustrate the approximate range when Pi is replete; however the exact boundary, or the exact time of onset of Pi starvation, has not been determined. Solid, dashed and dotted lines show the growth curves of RU1878, RU1879 and RU1880, respectively. Fluorescent bacteria populations are shown as mean ± SD from eight individual cultures for one starvation (C) or multiple consecutive starvations (D). For consecutive starvations, fluorescent populations at 150 min after each round of inoculation into media containing 50 mM Pi were used for the plot.

Effects of steady-state PhoB levels on fitness were analyzed in continuous cultures that maintain steady growth under Pi-depleted conditions. Similar to data from batch cultures shown in Figure 5B, the PhoB expression level of the repression mutant *P*_*phoB-rp2*_ is ~2 fold that of the corresponding WT (*P*_*phoB*_) in continuous cultures (Figure 6A). To assess cell fitness, all bacteria were transformed with a CFP-expressing plasmid and competed against a WT strain that expresses a non-fluorescent CFP variant. Fitness was evaluated by comparing the fluorescent bacteria population after 24 hr of competition against the population before competition. A population ratio of 1 was observed for the WT *P*_*phoB*_ (Figure 6B), indicating equal fitness for WT cells expressing CFP or the non-fluorescent CFP variant. The repression mutant *P*_*phoB-rp2*_ showed a ratio much lower than 1, suggesting that *P*_*phoB-rp2*_ is less fit than WT and thus was out-competed by the non-fluorescent WT strain.

Lowering the autoregulatory promoter strength of *P*_*phoB-rp2*_ could potentially decrease the steady-state level of PhoB to the WT level and recover the fitness loss. We mutated the 10 region of the *phoB-rp2* promoter and screened for mutants with weak promoter strength that gave comparable fluorescence output as the WT *phoB-yfp* reporter (Figures S5C and S5D). The selected clone, *phoB-rp2-dn*, showed no repression at high PhoB levels, similarly to *phoB-rp2*, but displayed low reporter output that is consistent with a low promoter strength (Figure S5E). The corresponding autoregulatory variant, *P*_*phoB-rp2-dn*_, expressed PhoB at ~90% of the WT level in continuous cultures under Pi-depleted conditions, much lower than that of *P*_*phoB-rp2*_ (Figure 6A). Fitness of this variant is higher than that of *P*_*phoB-rp2*_ (Figure 6B), suggesting that decreasing the steady-state PhoB expression to the WT level is beneficial to cells in continuous cultures.

As demonstrated by our model, lowering the promoter strength places a limit on PhoB expression speed and delays the response kinetics. This delay may impact cellfitness during the Pi starvation process. We performed bacterial competition assays in batch cultures when cells were transitioned from Pi-replete to Pi-depleted conditions. All strains have similar growth curves (Figure 6C, upper panel). When they were competed against the non-fluorescent WT, the strain with a low promoter strength, *P*_*phoB-rp2-dn*_, showed a slight decrease of population (Figure 6C, lower panel). After seven consecutive Pi starvation events, the decrease of population gradually accumulated to a significant level (Figure 6D), indicating a fitness disadvantage for *P*_*phoB-rp2-dn*_. In contrast, *P*_*phoB-rp2*_ and the WT *P*_*phoB*_, the two strains that have high promoter strength and fast PhoB expression kinetics, maintained an almost constant population throughout the repeated competitions. High steady-state concentration of PhoB in *P*_*phoB-rp2*_ appears to have little or no effect on cell fitness in batch cultures repetitively transitioned between Pi-replete and Pi-deplete conditions. Moreover, the WT strain expressing the non-fluorescent CFP variant displayed a constant population when competing against the WT strain expressing the fluorescent protein, suggesting that expression of fluorescent CFP has little impact on cell fitness.

In summary, continuous and batch cultures appear to have different fitness preferences. The steady Pi-depleted condition in continuous cultures favors cells with the optimal steadystate concentrations of PhoB. Dynamic changes in Pi concentrations during Pi starvation in batch cultures favor cells with fast response kinetics. An autoregulatory system with only positive feedback may not satisfy both requirements due to the intrinsic trade-off between steady-state level and response kinetics. The coupled negative feedback in WT resolves the conflict and achieves high fitness across different growth conditions.

## DISCUSSION

Feedback regulation is a common regulatory strategy that underlies a wide variety of signaling pathways (Alon, 2007; Groisman, 2016; Mitrophanov and Groisman, 2008). Many experimental studies have focused on various extraordinary behaviors generated by feedbacks, such as memory, oscillation, and bimodal responses, while the quantitative details of response fine-tuning during feedback regulation are often explored in engineered regulatory circuits or by mathematic modeling. Understanding a particular regulatory feature quantitatively in a naturally occurring system and determining its fitness gain/cost experimentally are usually challenging because a large number of signaling components need to be examined under cellular conditions. Built upon previous cellular characterization of the PhoB/PhoR system (Gao and Stock, 2013a, 2013b), we are able to measure the activation dynamics and quantitatively assess the cost of positive autoregulation on response speed. We discovered a coupled negative feedback that fine-tunes the response dynamics and reconciles different fitness requirements in different environments. Dynamic environments favor fast response kinetics while relatively static environments prefer optimal steady-state levels of TCS proteins, which may reflect requirements of the biphasic lifestyle of *E. co*li in both host and open environments. The coupled negative and positive feedbacks may have been evolved to give cell fitness advantages when establishing new growth during environmental transitions as well as maintaining steady growth in stable environments.

### Response Delay Determined by Design Features of Positive Autoregulation

An inherent response delay has long been recognized for positively autoregulated systems (Hermsen et al., 2011; Maeda and Sano, 2006; Mitrophanov and Groisman, 2008; Savageau, 1974). Although a delay of response can be advantageous in some systems (Mruk et al., 2007; Rosenfeld and Alon, 2003), it is usually detrimental to prompt signaling and needs to be either tolerated or circumvented, thus placing constraints on the design of regulatory circuits (Hermsen et al., 2011).

Our model reveals three major factors, RR phosphorylation kinetics, TF binding affinity, and promoter strength that shape the response time of gene expression controlled by a positively autoregulated RR transcription factor. In the PhoB-PhoR system, RR phosphorylation occurs relatively rapidly and RR transcription appears to be a rate-determining step in response speed. As demonstrated by the activation dynamics of the autoregulatory variant *P*_*phnC*_, the rising time of RR expression can be severely prolonged by a weak TF affinity for the promoter. A timely response requires a strong binding affinity to the autoregulated promoter, which is consistent with the discovery that phoB is expressed earlier than most PhoB-regulated promoters and the affinity for the autoregulated *phoB* promoter is among the highest within the PhoB regulon (Gao and Stock, 2015). Early activation and high conservation to the consensus binding sequence, a sign of strong affinity, have been observed for many autoregulated TCS promoters, such as, *phoP* in Salmonella enterica serovar Typhimurium (Zwir et al., 2012, 2014), *bvgA in Bordetella pertussis* (Karimova and Ullmann, 1997), *glnA* (regulated by NtrC) (Atkinson et al., 2002), and *cpxR* in *E. coli* (De Wulf et al., 2002). Strong affinity for the autoregulated promoter may represent a common design principle for positively autoregulated TCSs to reduce the inherent response delay. For systems in which a response delay is advantageous, the promoter affinity can be further fine-tuned for the desired temporal response.

Promoter strength affects response speed as well as steadystate expression levels of TCSs. Optimal steady-state levels of TCS proteins are correlated with the concentration-dependent RRP output profiles, which are determined by intrinsic TCS enzyme activities (Batchelor and Goulian, 2003; Gao and Stock, 2013a, 2013b). Different optimal RR concentrations require different promoter strengths for the basal and stimulated states. There is a fundamental trade-off between response speed and the auto-activated RR expression level. It has been predicted that the relative difference or the fold change between the basal and auto-activated TF expression levels need to be small or moderate to ensure a reasonably rapid induction time (Hermsen et al., 2011). Similarly, our quantitative model describes a response time limit constrained by steady-state expression levels of RR transcription factors. If no additional translational regulation is present, response time is limited by the maximal RR transcription rate, which is determined by the autoregulated promoter strength. A strong promoter leads to a fast response, with little cost for response speed but potentially with a substantial cost associated with high TF expression levels. Individual TFs have different expression levels as well as different fitness dependence on expression levels (Keren et al., 2016), thus there may not exist a universal mechanism to balance different costs for individual autoregulated systems. For the PhoB-PhoR system, the optimal fitness achieved at given steady-state concentrations of RR requires certain promoter strengths at the basal and stimulated states, which sets a rising time limit at ~60 min for a classical positively autoregulated system. The discovery of a shorter rising time for WT led to the uncovering of a coupled negative feedback that accelerates the response.

### Response Fine-Tuning by the Coupled Feedback System

Response acceleration by negative feedbacks has been described in many systems (Rosenfeld et al., 2002; Svenningsen et al., 2008; Teng et al., 2012). It has also been postulated that negative autoregulation coupled to auto-activation can attenuate the inherent response delay caused by positive feedback (Hermsen et al., 2011). Our study reveals the effectiveness of such a regulatory strategy that has been naturally evolved to circumvent the trade-off between response speed and expression level.

The capability of the PhoB-PhoR autoregulatory system to achieve both fast response speed and optimal expression is based upon different binding affinities to the activation and repression sites within the autoregulated promoter. A high-affinity activation site allows a fast response at low TF levels, while the low-affinity site enables repression at high TF levels and ensures that expression of the TF does not exceed the optimal level. This strategy offers great flexibility in fine-tuning the response speed as well as the expression level. Similar patterns of strong activation and weak repression sites have also been observed in other autoregulated TCS promoters, such as phoBR in *Vibrio cholerae* (Diniz et al., 2011) and *glnALG in E. coli* (Atkinson et al., 2002). Although the mechanism of transcription repression appears to be different as suggested by different positions of repression sites (Diniz et al., 2011; Wang et al., 2016), weak repression sites function to similarly limit the maximal concentration at high TF levels. Dual autoregulatory TFs constitute a non-trivial fraction (~10%) of autoregulated TFs in *E. coli* (Martínez-Antonio et al., 2008), and this number may even be underestimated due to difficulties in uncovering the weak autoregulatory interactions. It remains to be explored how many TFs share the mechanism described here to reconcile different fitness requirements.

Implementation of a coupled negative feedback may not be restricted to transcription auto-repression. For example, negative feedback can occur through negative regulation of the enzyme activity of an HK via ADP-stimulated RR phosphatase activity (Yeo et al., 2012) or expression of negative regulator proteins (Lippa and Goulian, 2009; Raivio et al., 1999; Salazar et al., 2016). Such negative feedback in the positively autoregulated PhoP-PhoQ system is suggested to promote an “overshoot” or impulse response, in which the response increases rapidly to a high level before decaying to an intermediate steady level (Ray and Igoshin, 2010; Salazar et al., 2016; Yeo et al., 2012). The overshoot dynamics can also potentially offset the intrinsic delay by positive autoregulation but may have a more sophisticated role in coupled feedback systems and requires further investigation. A minor overshoot of PhoB phosphorylation kinetics has been observed only in the engineered constitutive strain (Figure S1C), but not in WT bacteria (Figure 3A). This suggests that auto-repression of the *phoB* promoter does not promote the impulse response and another yet unknown negative feedback may operate in the engineered system.

Coupled feedbacks have been extensively studied for their ability to fine-tune bistable responses or elicit oscillations (Avendaño et al., 2013; Kim et al., 2008; Tiwari and Igoshin, 2012). Neither behavior has been observed in the WT PhoB-PhoR system. Even without the complex and extraordinary bistable or pulse responses discovered in a few other TCSs (Levine et al., 2012; Narula et al., 2015; Tiwari et al., 2011), the fine details of temporal dynamic responses and the steady state can be modulated by the coupled feedbacks to give cells optimal fitness. Protein expression dynamics are usually tightly coupled with steady-state behaviors, making it difficult to assess the role of one feature without interfering with the other and shaping the fundamental trade-off between response speed and expression levels. The coupled feedbacks not only overcome the intrinsic response speed limit but also allow a wide range of response dynamics with similar steady-state expression levels. Such feedbacks could be used to engineer systems with variations in response dynamics to investigate how a single dynamic feature impacts cell fitness.

## STAR⋆METHODS

### KEY RESOURCES TABLE

**Table:** 

REAGENT or RESOURCE	SOURCE	IDENTIFIER
Antibodies		

Rabbit polyclonal anti-PhoB serum	This study	RRID: AB_2722768
Cy5 Goat anti-rabbit IgG (H+L)	ThermoFisher	Cat# A10523; Lot #1512070; RRID: AB_2534032

Bacterial and Virus Strains		

*E. coli* DH5a [general cloning strain]	ThermoFisher	Cat# 18265017
*E. coli* GM2929 [*dam^−^ dcm^−^*, cloning strain]	CGSC, Yale University	CGSC# 7080
*E. coli* BW25113 [wild type]	Datsenko and Wanner, 2000	N/A
*E. coli* BW25141 [*pir* D*phoBR580*, cloning strain for CRIM integration plasmids]	Haldimann and Wanner, 2001	N/A
*E. coli* RU1616 [LAC, Φ(D*phoBp* P_lac_-*phoBR*) in BW25113]	Gao and Stock, 2013b	N/A
*E. coli* RU1621 [Δ*phoBR* in BW25113 derivative]	Gao and Stock, 2013b	N/A
*E. coli* RU1646 [Δ*rpoS*::kan in BW25113]	Gao et al., 2017	N/A
*E. coli* RU1783 [TRC*, F(Δ*phoBp* P_trc_-*phoBR*) *attl*::pRG378(lacI^q^)]	Gao and Stock, 2015	N/A
*E. coli* RU1878 [P_phoB_-phoBR, attHK::pRG390 in RU1621]	This paper	N/A
*E. coli* RU1879 [P_phoB-rp2_-phoBR, attHK::pRG391 in RU1621]	This paper	N/A
*E. coli* RU1880 [P_phoB-rp2-dn_-phoBR, attHK::pRG393 in RU1621]	This paper	N/A
*E. coli* RU1881 [P_phnC_-phoBR, attHK::pRG396 in RU1621]	This paper	N/A

Chemicals, Peptides, and Recombinant Proteins		

Phos-tag Acrylamide AAL-107	Wako Chemicals	Cat# 304–93521
*E. coli* PhoB protein	Gao and Stock, 2015	N/A

Oligonucleotides		

RG278-f: TCTGACACATAATGACGTCGCA	This paper	RG278-f
RG279-r: TGCGACGTCATTATGTGTCAGA	This paper	RG279-r
RG280-f: ATCTGTTCCATAATGTGCTCGCATTA	This paper	RG280-f
RG281-r: TAATGCGAGCACATTATGGAACAGAT	This paper	RG281-r
RG282-f: TCTGTTCCATAATGACGTCGCATTA	This paper	RG282-f
RG283-r: TGCGACGTCATTATGGAACAGATTTATGAC	This paper	RG283-r
RG293-r:TTGCGATCATTAATGCGAGCACATNATN GAACAGAT	This paper	RG293-r
RG294-f: GTGCTCGCATTAATGATCGCAACC	This paper	RG294-f
RG322-f: TGACCACCCTGGCCTCGGCCGTGCAGT GCTTCA-3	This paper	RG322-f
RG323-r: AGCACTGCACGGCCGAGGCCAGGGTG GTCACGA	This paper	RG323-r

Recombinant DNA		

pAH144 [CRIM plasmid for integration at *attHK* site, Sp^r^]	Haldimann and Wanner, 2001	pAH144
pRG22 [Multi-cloning site (MCS) in a p15A orgin plasmid, Cm^r^]	Mack et al., 2009	pRG22
pJZG146 [*rrnB*-MCS-*mYFP*, Sp^r^]	Gao and Stock, 2013a	pJZG146
pRG161 [*PphoA-mYFP* in pJZG146, Sp^r^]	Gao and Stock, 2013a	pRG161
pJZG202 [*PphoB-mYFP* in pJZG146, Sp^r^]	Gao and Stock, 2015	pJZG202
pRG162 [*PphnC-mYFP*in pJZG146, Sp^r^]	Gao and Stock, 2015	pRG162
pRG252 [*Ptet* promoter, Ap ^r^]	Gao and Stock, 2013a	pRG252
pRG367 [*PphoB-rp-hi-mYFP* in pJZG146, Sp^r^]	This paper	pRG367
pRG368 [*PphoB-rp2-mYFP* in pJZG146, Sp^r^]	This paper	pRG368
pRG369 [*PphoB-rp1-mYFP* in pJZG146, Sp^r^]	This paper	pRG369
pRG387 [*PphoB-rp2-dn-mYFP* in pJZG146, Sp^r^]	This paper	pRG387
pRG390 [*P_phoB_-phoBR* in pAH144, Sp^r^]	This paper	pRG390
pRG391 [*P_phoB-rp2_-phoBR* in pAH144, Sp^r^]	This paper	pRG391
pRG393 [*P_phoB-rp2-dn_-phoBR* in pAH144, Sp^r^]	This paper	pRG393
pRG396 [*P_phnC_-phoBR* in pAH144, Sp^r^]	This paper	pRG396
pRG411 [*Ptet-CFP* in pRG22, Cm^r^]	This paper	pRG411
pRG426 [*Ptet-CFP(T65A W66S G67A)* in pRG22, Cm^r^]	This paper	pRG426
*phoB* promoter DNA [for EMSA]	This paper	N/A
*phnC* promoter DNA [for EMSA]	This paper	N/A

Software and Algorithms		

MATLAB R2009a	MathWorks	https://www.mathworks.com/
OriginPro 8	OriginLab	https://www.originlab.com/
FIMO tool (MEME suite)	Bailey et al., 2009	http://meme-suite.org/tools/fimo
RSAT tool	Medina-Rivera et al., 2015	http://embnet.ccg.unam.mx/rsat/
ImageJ	Schneider et al., 2012	https://imagej.nih.gov/ij/
Thresholding algorithm for colony counting	This paper	N/A

### CONTACT FOR REAGENT AND RESOURCE SHARING

Further information and requests for resources and reagents should be directed to and will be fulfilled by the corresponding author Ann M. Stock (stock@cabm.rutgers.edu).

### EXPERIMENTAL MODEL AND SUBJECT DETAILS

#### *E. coli* strains and plasmids

Bacterial strains and plasmids used in this study are listed in the Key Resources Table. All strains used for *in vivo* assays were derived from BW25113 (Datsenko and Wanner, 2000). Autoregulatory variants with altered promoters for phoBR were incorporated in the chromosome at the HK022 phage attachment site using the CRIM recombination strategies (Haldimann and Wanner, 2001).

#### Bacterial growth conditions

Bacteria were grown at 37°C in LB broth or MOPS minimal media (Neidhardt et al., 1974) with 0.4% (w/v) glucose and amino acid mix (40 mg/ml). Specifically, MOPS minimal media containing 40 mM MOPS, 4 mM Tricine, 50 mM NaCl, 0.276 mM K_2_SO_4_, 0.523 mM MgCl_2_, 0.01 mM FeSO_4_ and other micronutrients were made as described (Gao et al., 2017). Plasmid maintenance was accomplished by adding carbenicillin at 100 μg/ml, chloramphenicol at 34 μg/ml or spectinomycin at 30 μg/ml. For batch cultures, the MOPS media contained 5 mM NH_4_Cl and amino acid mix (40 μg/ml) as the nitrogen source. For continuous cultures, amino acids were not added and the concentration of NH_4_Cl was reduced to 250 μM to limit the culture growth (Gao and Stock, 2013a). Phosphate concentration in the feed media was set at 12 μM to ensure a Pi-depleted condition in the culture tube (Pi < 1μM) (Gao and Stock, 2013a). The dilution rate was set at ~0.25 h^−1^ by controlling the feed media flow rate at ~6 ml/h and the total culture volume at 24 ml. Bacteria from fresh MOPS cultures were used for inoculation with a starting OD (600 nm) of ~0.04 and the steady OD of the continuous culture were ~0.08.

### METHOD DETAILS

#### Cloning of strains and plasmids

To generate promoter mutants with altered PhoB repression sites, the following primers were used for site-directed mutagenesis with pJZG202 as PCR template: RG278-f and RG279-r for *phoB-rp-hi*; RG280-f and RG281-r for *phoB-rp2*; RG282-f and RG283-r for *phoB-rp1*. Recombinant PCR fragments were digested with PstI/XbaI and cloned into pJZG202 to give pRG367, 368 and 369. Individual promoter fragments corresponding to *PphoBwt* and *PphoB-rp2* were then excised and ligated with a promoter-less *phoBR* fragment followed by insertion into SphI/SmaI-digested pAH144 to give pRG390 and pRG391.

To screen for *phoB-rp2* variants with reduced promoter strength, primer RG294-f and the degenerate primer RG293-r were used to generate recombinant PCR fragments with random mutations at two positions in the 10 region of *phoB* promoter. The resulting DNA fragments were ligated into the PstI and XbaI sites of pJZG202 and screened for reporter activities that were comparable to that of the WT *phoB* promoter. One such clone with a sequence of CATGAT at the 10 region was selected and designated as pRG387. PCR fragments containing either the phnC promoter or the promoter region of pRG387 were fused with phoBR by recombinant PCR and inserted into the BsrGI and SphI sites of pRG390 to give pRG396 and pRG393. The four integration plasmids, pRG390, 391, 393 and 396, were integrated into the chromosome of RU1621 at the HK022 phage attachment site (Haldimann and Wanner, 2001) to create RU1878 (*phoBwt*), RU1879 (*phoB-rp2*), RU1880 *(phoB-rp2-dn)* and RU1881 (*phnC).*

To evaluate cell fitness in bacterial competition assays, a DNA fragment containing *Ptet-cfp* was excised from a pRG252-derived plasmid and inserted into pRG22 to give pRG411, the CFP-expressing marker plasmid. Primers RG322-f and RG323-r were used to introduce site-specific mutations into the *cfp* gene of pRG411. The resulting plasmid pRG426 has a non-fluorescent CFP protein (CFP T65A W66S G67A) expressed from the *Ptet* promoter, enabling the differentiation of fluorescent cells that carry pRG411.

#### Phosphate starvation

Cells from overnight MOPS batch cultures were used to inoculate fresh Pi-replete (1 mM KH_2_PO_4_) MOPS media. Once the OD reached 0.3–0.5, bacteria were harvested and washed with MOPS medium (30–50 μM Pi, non-activating) twice and directly resuspended in MOPS medium (2 μM Pi, activating) for Pi starvation. The starting OD was typically between 0.1–0.2. To enable simultaneous PhoB induction and Pi starvation shown in Figure 1, 150 μM IPTG was added only to the starvation medium (2 mM Pi) and not to the Pi replete MOPS media. On the other hand, to achieve different constant PhoB expression levels for the non-autoregulatory strains, different IPTG concentrations were included in all the culture media throughout the assay (Gao and Stock, 2013b, 2015). For RU1616, IPTG concentrations of 0, 5, 15, 25, 50, 75 and 150 μM were used to generate PhoB expression levels at 0.4, 0.9, 1.7, 2.4, 4.8, 6.5 and 8.2 μM. For RU1783, IPTG concentrations of 0, 1.5, 5 and 15 μM were used to yield PhoB levels at 4.3, 7.6, 11.2 and 23.5 μM. Inoculated cultures were transferred to 96-well plates for reporter assays while aliquots from similarly prepared bulk cultures were removed at indicated time points for analyses of PhoB expression and phosphorylation kinetics. Approximately 0.3 OD,ml of cell pellets were used for the subsequent quantitative western analyses and Phos-tag analyses.

#### Determination of PhoB expression and phosphorylation levels

PhoB expression and *in vivo* phosphorylation levels were measured using previously established procedures (Gao and Stock, 2013b). Specifically, ~0.3 OD ml of bacteria pellets were prepared as above. For PhoB expression analyses, pellets were lysed by boiling in 70 μL of 1xSDS sample loading buffer (62.5 μM Tris pH 6.8, 2% SDS, 10% glycerol and 0.05% bromopenol blue). For phosphorylation analyses, cell pellets were lysed by repeated pipetting up and down for ~10 s in 55 μL of 1x BugBuster reagent (Novagen) in 50 mM Tris, 100 mM NaCl, pH7.4 with 0.1% (v/v) Lysonase reagent (Novagen) followed by addition of 18 μL of 4x SDS loading buffer. Lysates were frozen immediately in a dry ice/ethanol bath and later analyzed using standard 15% SDS-PAGE or 10% Phos-tag gels. Proteins on gels were subsequently transferred to nitrocellulose membrane (0.45 μm pore size, GE Healthcare) at 75 mA per membrane for 75 min (standard gel) or 150 mA per membrane for 120 min (Phos-tag gel) using a Trans-Blot SD semi-dry transfer cell (Bio-Rad).

Western blotting was performed with a standard protocol using 5% non-fat milk as the blocking agent, rabbit polyclonal anti-PhoB C-terminal domain sera (1:1500) as the primary antibody and a Cy5-conjugated goat anti-rabbit IgG (1:5000) as the secondary antibody. Fluorescent blots were visualized using a FluorChem Q imager (Alpha Innotech) and quantified by ImageJ (Schneider et al., 2012). For PhoB~P levels, the fraction of PhoB proteins that are phosphorylated were calculated as the intensity ratio of the PhoBP band to the total of both PhoB and PhoB~P bands. Because PhoB~P levels were analyzed in strains expressing PhoB at constant concentrations that have been determined previously (Gao and Stock, 2013b, 2015), multiplying the PhoB~P fractions with total PhoB concentrations gives the PhoBP concentrations at various time points. To determine PhoB expression kinetics for different autoregulatory strains, the steady-state PhoB expression sample, i.e., the lysate sample for either BW25113(WT) or RU1878 (*phoBwt*) after 120 min of Pi starvation, was used as a reference sample in each blot for comparison to all other samples. The steadystate PhoB concentration of the WT strain has been determined previously (Gao and Stock, 2013b), thus allowing the calculation of PhoB levels for different samples.

#### Fluorescence reporter assays

Inoculated cultures in 96-well plates were continuously assayed for YFP fluorescence (excitation 488 nm, emission 530 nm) and OD 600 nm using a Varioskan plate reader (Thermo Scientific) with constant shaking. Fluorescence and OD readings were smoothed with a moving average of three time points for further analyses. First derivatives of fluorescence (dFluo./dt) were calculated numerically as described (Gao and Stock, 2017) by differentiating the 2^nd^ order Lagrange interpolating polynomial using the following equation,

$$f^{\prime }\left(t_{i}\right)=\frac{t_{i}-t_{i+1}}{\left(t_{i-1}-t_{i}\right)\left(t_{i-1}-t_{i+1}\right)}f\left(t_{i-1}\right)+\frac{2t_{i}-t_{i-1}-t_{i+1}}{\left(t_{i}-t_{i-1}\right)\left(t_{i}-t_{i+1}\right)}f\left(t_{i}\right)+\frac{t_{i}-t_{i-1}}{\left(t_{i+1}-t_{i-1}\right)\left(t_{i+1}-t_{i}\right)}f\left(t_{i+1}\right)$$

in which *f (t*_*i*_*)* represents the fluorescence at the *i*^*th*^ time point. The first derivatives were normalized to the OD to represent the promoter activity [(dFluo./dt)/OD].

To analyze the dependence of reporter activities (Fluo./OD) on PhoB expression levels (Figures 3D, 4D, S4, and S5), a slightly different starvation protocol was used to allow the data to be compared to prior similar analyses (Gao and Stock, 2015). A starting Pi concentration of 50 mM (non-activating), instead of 2 mM, was used in starvation assays, thus the starting time of the assay is not the onset time of Pi starvation. The starvation onset time was identified as the time point when fluorescence accumulation accelerates and the second derivative of fluorescence peaks (Gao and Stock, 2015). Reporter activities 90 min after the onset of starvation were arbitrarily chosen for comparison across different PhoB expression levels.

#### PhoB-binding site scanning

A total of 21 *PhoB*-binding sites (Gao and Stock, 2015) have been used to generate a 22-bp matrix to scan for potential binding sites. The *E. coli phoB* promoter sequence corresponding to 300 to +50 relative to the start codon of *phoB* was searched using the FIMO tool of the MEME suite (Bailey et al., 2009). One additional site overlapping the originally identified PHO box was discovered. It is a half-site adjacent to the downstream half-site of the original PHO box. This prompted us to search for half-sites with flexible spacing instead of the full site that has a fixed number of spacing nucleotides. We searched the same promoter sequence with the motif matrix generated with the 42 half-sites. The RSAT tool (Medina-Rivera et al., 2015) was used to identify weak half-sites that are adjacent to each other. Two half-sites overlapping by 1 bp were discovered on the antisense strand and chosen for further analyses.

#### Electrophoretic mobility shift assays

DNA fragments labeled with 5^’^-fluorescein (FAM) were used for EMSA with phosphorylated PhoB proteins as described previously (Gao and Stock, 2015). RG-FAM-1: 5^0^-GCTCACCA TTTGTATATCTCCTTC was used in combination with other promoter-specific primers to generate fluorescent DNA fragments containing either the *phoB* or *phnC* promoters. PCR products were purified with QIAquick columns (QIAGEN) and used for subsequent EMSA assays. For binding analyses of the *phoB* promoter repression site, fluorescein-labeled oligos were used instead of the full length of the promoter. DNA concentrations were determined by absorbance reading at 260nm using Nanodrop. DNA sequences used for EMSA are shown below with PhoB-binding sites underlined and lower case letters indicating common DNA sequences from the reporter vector:

#### phnC

CTGCAGGAAGAAGGAAAACGCTGGTTTGACAATCTTGCCGCTAACGGAAAAATCGAAATGGCCTGGCAGGAAACTTTCTGGGCGC ATGGCTTTGGCAAAGTCACCGATAAATTTGGCGTACCGTGGATGATTAATGTCGTCAAACAACAACCAACGCAATAACCCGCCGGG AGGCCCGCCCTCCCGCACTGTCATCGAATTCCCGTTAACTCTTCATCTGTTAGTCACTTTTAATTAACCAAATCGTCACAATAATCCG CCACGATGGAGCCACTTTTTTAGGGAGGCTGCATCATGCAAACGATTtgatctagaaataattttgtttaactttaagaaggagatatacaaatggtgagc

#### phoB

GCCACGGAAATCAATAACCTGAAGATATGTGCGACGAGCTTTTCATAAATCTGTCATAAATCTGACGCATAATGACGTCGCATTAAT GATCGCAACCTATTTATTACAACAGGGCtgatctagaaataattttgtttaactttaagaaggagatatacaaatggtgagc

#### *phoB-rp* (Repression site of phoB promoter, antisense strand)

5^’^-FAM- AATGCGA CGTCA TTATG CGTCA GATTTAT

#### *phoB-rp2* (Repression site mutant)

5^’^-FAM- AATGCGA GCACA TTATG GAACA GATTTAT

The protein-DNA binding buffer contained 50 mM Tris, pH 7.6, 200 mM NaCl, 0.1 mg/ml bovine serum albumin, 2 mM MgCl_2_, 1 mM dithiothreitol and 5% glycerol. Purified PhoB protein (~35 μM) was phosphorylated with 50 mM phosphoramidate for at least 1.5 h and different concentrations of PhoBP were subsequently incubated with approximately 0.1 mM fluorescent DNA for 30 min in the presence of 15 μM non-fluorescent competitor dsDNA oligos. PhoB-bound DNA complexes were analyzed by 12% TBE gels (130 v, 50 min on ice) and visualized by fluorescence imaging using a FluorChem Q imager. The fraction of bound DNA was calculated based on quantification of DNA band intensities using ImageJ. Binding curves were generally fitted with the Hill equation (OriginPro 8) to derive the dissociation constant *K*_*D*_. For DNA fragments that have a relatively strong affinity with *K*_*D*_ close to the experimental DNA concentration, specifically, the *phoB* promoter, protein bound to DNA causes a non-trivial reduction in the concentration of free PhoB~P thus the total concentration of PhoB~P cannot be used directly for curve fitting. We assumed a two-site per DNA-binding model and used the Simbiology tool of MATLAB to estimate the *K*_*D*_. The estimated *K*_*D*_ is 0.25 μM, about one seventh the K_D_ of *phnC*, which is consistent with the difference in off-rates measured previously (Gao and Stock, 2015).

#### Mathematic model of PhoB autoregulation

Kinetics of PhoB autoregulation were simulated with the Simbiology tool of MATLAB. Parameter values are listed in Table S2. A deterministic model was employed with two major regulatory modules: (i) the phosphorylation cycle that determines the output PhoBP concentration by various kinase and phosphatase activities of PhoR, and (ii) the transcription feedback that controls the expression of PhoB and PhoR proteins.

The phosphorylation cycle was modeled similarly to that described previously (Batchelor and Goulian, 2003; Gao and Stock, 2013b; Siryaporn et al., 2010). For given PhoB and PhoR levels, phosphorylation kinetics of PhoB are regulated by various enzyme activities of PhoR and PhoB. Four major enzyme reactions were considered (Figure S1): (i) autophosphorylation and dephosphorylation of PhoR, (ii) phosphotransfer from PhoR~P to PhoB, (iii) dephosphorylation of PhoB~P by PhoR and (iv) autodephosphorylation of PhoB~P. Autophosphorylation of PhoR was approximated with first order kinetics considering that the cellular ATP concentration is in large excess. Mass-action kinetics were used to model the binding of PhoB or PhoBP to PhoR and the subsequent phosphotransfer or dephosphorylation reactions. We examined in vivo phosphorylation kinetics of PhoB at three different constant PhoB levels, however the data were not sufficient to derive the values for all nine parameters describing the above reactions. Based on previous *in vitro* analyses (Gao and Stock, 2013b), the rate constant of PhoB autodephosphorylation, k_y_, was set at 2.6 ×10^4^ s^−1^. Binding rates *k*_*1*_ and *k*_*2*_ were assumed to be diffusion-limited with rate constant 0.15 μM^−1^s^−1^ while binding affinities for the phosphotransfer and phosphatase complexes were assumed to be equal. This allows the scanning and estimation of other parameters to generate phosphorylation kinetic curves comparable to experimental data (Figure S1).

PhoB~P concentration is the input to transcription control that determines the total concentration of PhoB and PhoR in the cell. The positive autoregulation of PhoB expression was described with the equation shown in Figure 2. Because PhoB~P binds DNA as a dimer (Blanco et al., 2012; Ritzefeld et al., 2013), the Hill coefficient n was set at 2. DNA-binding affinities of PhoB~P determined by in vitro EMSA experiments were discovered to be within the same magnitude as those determined by *in vivo* reporter assays (Gao and Stock, 2015) and thus were used for modeling. Repression of the *phoB* promoter was modeled by multiplying a repression factor as shown below:

$$\text{PhoB}\;\text{expression}\;\text{rate}=\left(P_{0}+P*\frac{{\left[PhoB\sim P\right]}^{n}}{{\left[PhoB\sim P\right]}^{n}+K_{DNA}^{n}}\right)*\left(1-\frac{{\left[PhoB\sim P\right]}^{2}}{{\left[PhoB\sim P\right]}^{2}+K_{RP}^{2}}\right)$$

in which K_RP_ represents the binding affinity of PhoB~P to the repression site of the phoB promoter.

We estimated the protein production rate constants *P*_0_ and *P* based on previous determinations of steady-state concentrations of PhoB. The value of *P*_*0*_ is based on the following equation with equal rates of protein production and protein decrease at steady state:

$$P_{o}=k_{dil}*{\left[phoB\right]}_{0}$$

in which [*PhoB*]_0_ is the basal PhoB level under the Pi-replete condition. The value of *k*_*dil*_ depends on the growth dilution rate as well as the PhoB degradation rate. PhoB is relatively stable over the time period investigated (Gao et al., 2017) thus we used the growth rate to estimate *k*_*dil*_. When cells have been Pi-depleted for a long time, cell growth is very slow. For simplicity, we did not consider this further growth rate reduction, nor the effect of the stress response, such as the RpoS effect, for the majority of modeling tasks. The initial growth rate at the early stage (15–45 min) of Pi starvation was used to estimate *k*_*dil*_ while both *P*_*0*_ and *k*_*dil*_ were kept constant throughout the modeled time course. For data in Figures S3E and S3F in which the growth rate reduction and the general stress response were considered, real-time growth rates at individual time points were calculated as OD-normalized first derivatives of OD and fitted with a Hill function. Both *P*_*0*_ and *k*_*dil*_ were allowed to decrease simultaneously, following the fitted Hill function and reflecting a general reduction of growth and transcription. As for the value of P, it was found that Pi-depletion increased the PhoB concentration ~21 fold (Gao and Stock, 2013b), therefore, a value of 20xP_0_ was used initially. For *phoB-rp2*, a value of 41x*P*_*0*_ was used to give a total PhoB induction of 42 fold, which is approximately two times that of the WT level observed for both reporter activity and PhoB expression (Figures 4E and 5B). The PhoR production rate was always set as one tenth the PhoB expression rate to maintain the observed PhoR:PhoB ratio of 1:10 (Gao and Stock, 2013b). Kinetics of PhoB~P and total PhoB concentrations were simulated with indicated parameter values using the ODE15s solver of MATLAB.

#### Evaluation of cell fitness by bacterial competition

Bacterial competition assays were performed in both continuous and batch cultures. Indicated strains carrying pRG411 expressing CFP were competed against RU1878 (*phoBwt*) carrying pRG426 expressing a non-fluorescent CFP variant. Continuous cultures were inoculated with mixed populations of bacteria and the same Pi-depleted condition as described in growth conditions was maintained. Bacterial cultures were sampled 24 h after inoculation and diluted 2500 fold before spreading on chloramphenicol-containing LB plates. At least two aliquots were plated for each sample. Bacterial colonies, ~500–1000 per plate, were visualized a day later by fluorescence imaging using a FluorChem Q imager. The numbers of total and fluorescent colonies were counted with ImageJ using an image-thresholding algorithm. Similar as the algorithm developed for single-cell imaging (Miyashiro and Goulian, 2007), the algorithm counts the number of particles on each image for a range of threshold values and the particle counts are then plotted against the threshold values. Two plateaus of counts at low and high threshold values correspond to the total number of colonies and the number of fluorescent colonies (see Figure S6 for details). The fraction of fluorescent colonies after the competition was compared to the fraction before the competition to assess the fitness of individual strains.

For batch culture competition, bacterial mixtures were inoculated in 96-well plates with a starting Pi concentration of 50 μM. Due to bacterial growth, Pi gradually decreases and the depletion of Pi elicits the starvation response. After 3 h, 50 μL of bacterial cultures were removed and inoculated into 225 mL of fresh MOPS (50 μM Pi) to start another round of starvation. Plates were stored at 4°C overnight every 2 or 3 rounds of starvation before inoculation for the next round. CFP fluorescence (excitation 430 nm, emission 475 nm) and OD were measured every 10 min during the competition. Pure populations of bacteria carrying pRG411 or pRG426 were grown simultaneously as controls. Basal fluorescence of bacteria carrying pRG426 was treated as background fluorescence and subtracted from the measured fluorescence. The population of fluorescent bacteria was calculated by dividing the OD-normalized fluorescence of the bacterial mixture with that of pure fluorescent cells that carry pRG411.

### QUANTIFICATION AND STATISTICAL ANALYSIS

In all analyses, mean values are presented and SDs are shown as error bars. The numbers of independent samples or replicates are reported in individual figure legends. Fluorescence reporter data were analyzed by Microsoft Excel and MATLAB r2009. Data fitting were performed using OriginPro8 (OriginLab).

Band intensities of western blots or EMSA gels were quantified with ImageJ 1.50i (NIH) using the gel analyzer tool to generate line graphs for all bands of interest. Relative band intensities were computed as relative peak areas for individual bands and subsequently compared to the calibration samples or the control samples with known quantities of protein for quantification. Details of control or calibration samples are documented in METHOD DETAILS.

## Supplementary Material

1

2

## Highlights

- Positive autoregulation of transcription carries fitness cost on response speed
- Requirements for optimal protein expression levels place limits on response speed
- Strong promoter accelerates response with a cost of increased protein levels
- Auto-repression allows fast response without increasing expression levels
