# Supplementary material for: Overcoming the Cost of Positive Autoregulation by Accelerating the Response with a Coupled Negative Feedback
Source: Cell Rep. Author manuscript; Available in PMC 2018 Oct 19. (PMC6194859; doi:10.1016/j.celrep.2018.08.023)
Supplement: 1 [file NIHMS1507859-supplement-1.pdf]

Cell Reports, Volume *24*

Supplemental Information

Overcoming the Cost of Positive Autoregulation by Accelerating the Response with a Coupled Negative Feedback

Rong Gao and Ann M. Stock

Figure S1

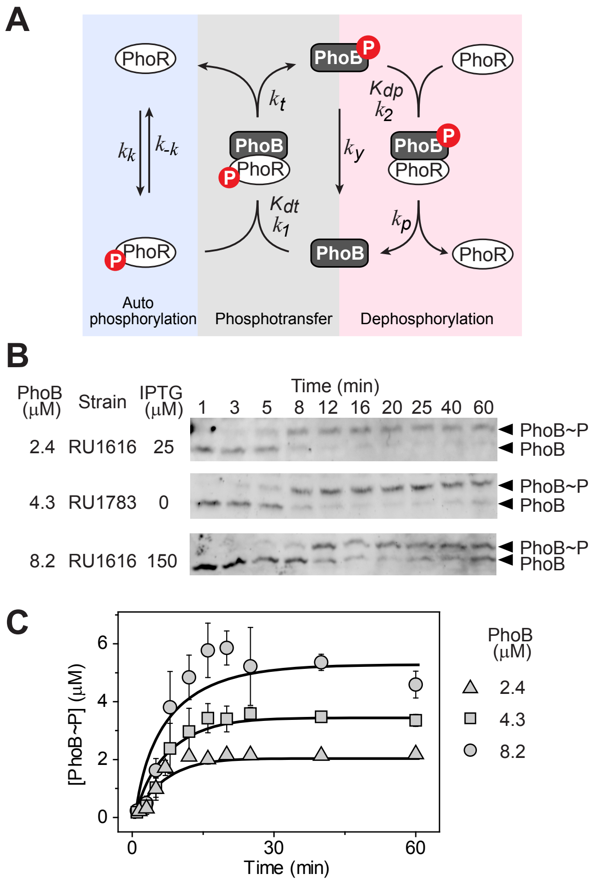

Figure S1. Parameter estimation for the phosphorylation reactions. Related to Figure 2.
(A) Reactions and parameters used to model the phosphorylation module. (B and C) *in vivo* phosphorylation kinetics of PhoB analyzed by immnoblotting of Phos-tag gels. Indicated PhoB levels were maintained in the non-autoregulatory strains and PhoB~P fractions were tracked upon Pi starvation. PhoB~P levels were calculated by multiplying the total PhoB concentrations with the PhoB~P fractions derived from Phos-tag gels. Error bars are SDs of at least three independent experiments and unseen error bars are smaller than symbol sizes. Parameters were estimated using Matlab to generate comparable phosphorylation kinetics curves (solid lines) at all three PhoB levels. Parameter values are listed in Table S1.

Figure S2

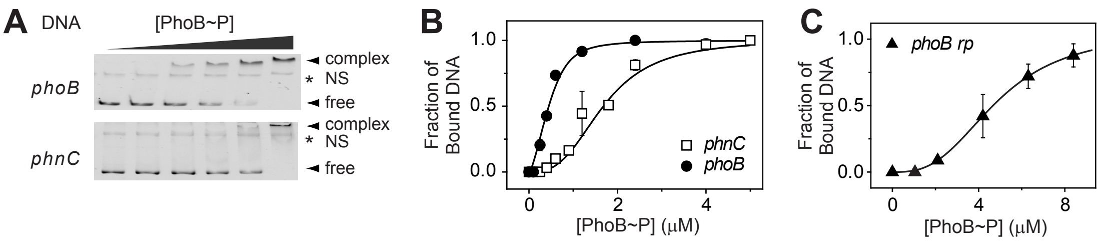

Figure S2. **Determination of the PhoB~P binding affinity with EMSA. Related to Figure 2.**
(A and B) Binding of PhoB~P to *phoB* and *phnC* promoter DNA. EMSA was performed using PCR-generated fluorescent DNA fragments in the presence of 0, 0.1, 0.25, 0.4, 0.6, and 2.4 μM PhoB~P. Asterisks indicate non-specific DNA binding (NS). One representative gel is shown to compare the binding of PhoB~P to *phoB* and *phnC*. Data in (B) are derived from two experiments with more PhoB~P concentrations than shown in (A). Solid lines indicate the fitted curves. Binding fractions for the *phnC* promoter were fitted with a Hill equation. Total concentrations of PhoB~P, instead of the free PhoB~P concentrations, were directly used for the fitting because the fraction of PhoB~P bound to DNA is relatively small due to the low affinity for *phnC*. For *phoB*, the fraction of bound PhoB~P is not negligible, especially at low PhoB~P levels. Thus, a two-site binding model was built in Matlab and used for data fitting. (C) Binding of PhoB~P to the repression site. EMSA data shown in Figure 4B were quantified and fitted with the Hill equation. Data are shown as mean ± SD from three experiments.

Figure S3

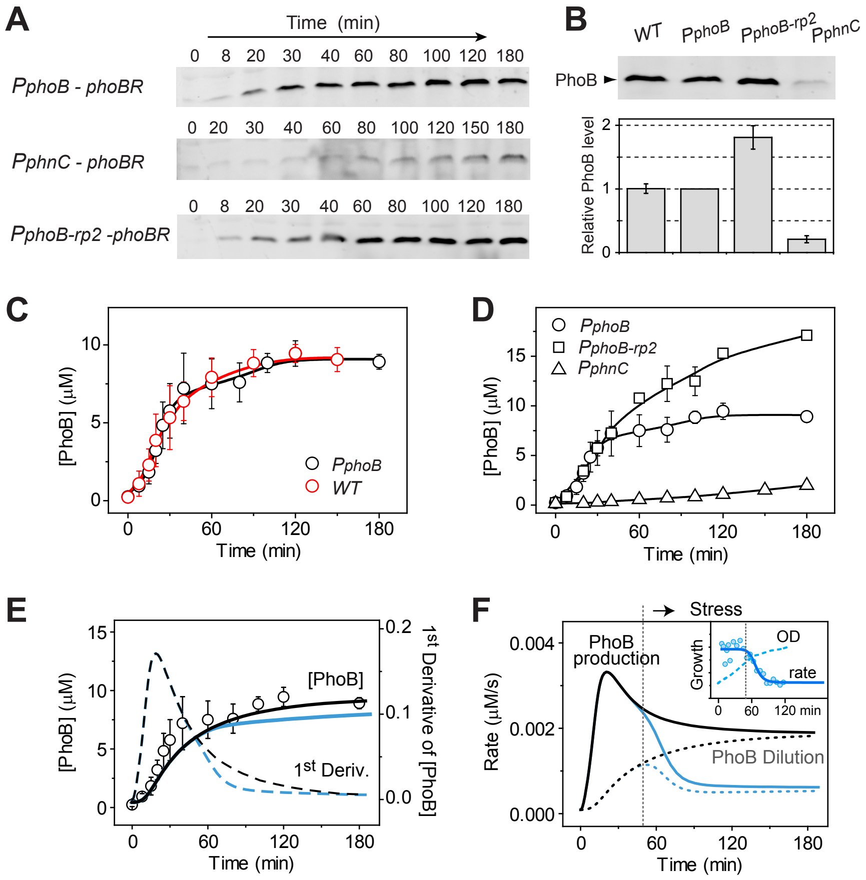

Figure S3. **Kinetics of PhoB expression for the autoregulatory variants. Related to Figure 2 & 5.**
(A) PhoB expression upon Pi starvation. Following Pi starvation in MOPS (2 µM Pi) media, PhoB expression was examined by immunoblotting in strains RU1878 (P_{phoB}), RU1879 ($P_{phoB\text{-}rp2}$) and RU1881 (P_{phnC}). (B) Comparison of PhoB expression levels. Final PhoB levels for strain BW25113 (WT) and the autoregulatory variants RU1879 and RU1881 were compared to that of RU1878. The sampled time and the number of samples for each strain were: WT, 120 min, n=3; P_{phoB}, 120 min, reference strain, number not applicable; $P_{phoB\text{-}rp2}$, 180 min, n=8; and P_{phnC}, 180 min, n=6. (C) Indistinguishable kinetics for the autoregulated strains BW25113 (WT) and RU1878 (P_{phoB}). Both strains contain the wild-type *phoBR* operon but the location of *phoBR* for RU1878 is at the HK022 phage attachment site instead of the original chromosomal position. (D) PhoB expression kinetics for the autoregulatory variants. Data in C and D are shown as mean ± SD from the following number of experiments: WT, n=8; P_{phoB}, n=5; $P_{phoB\text{-}rp2}$, n=3; and P_{phnC}, n=4. (E and F) PhoB concentration kinetics modeled with (blue lines) or without (black lines) the general stress response. Circles in (E) indicate PhoB concentrations measured experimentally for the strain RU1878. Solid lines represent kinetics that are modeled with the coupled negative feedback while dashed lines are the calculated first derivatives from the modeled data. (F) Rates of PhoB production (solid curves) and dilution (dotted curves). Approximately 50 min after Pi starvation (vertical dotted lines), the bacterial growth rate started to decrease and reached about one third of the initial growth rate (inset), resulting in the corresponding reduction of the dilution rate. Stress reponse is modeled with a general reduction of transcription rate similar to the growth rate reduction. It leads to further repression of PhoB production rate in addition to PhoB auto-repression.

Figure S4

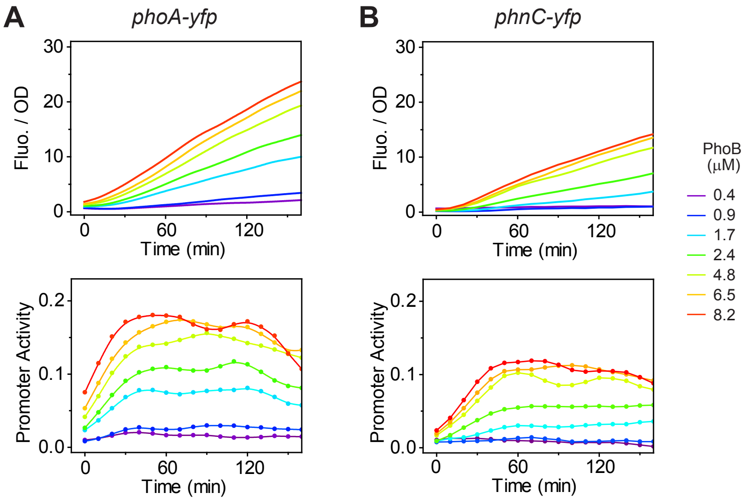

Figure S4. **Monotonic relationship between promoter activity and PhoB levels for the *phoA* (A) and *phnC* (B) promoters. Related to Figure 3.**

Fluorescent reporter dynamics were monitored for pRG161 (*phoA-yfp*) and pRG162 (*phnC-yfp*) in the non-autoregulatory strain RU1616 at the indicated PhoB levels. OD-normalized fluorescence (Fluo./OD) and the first derivative (promoter activity) are shown for one representative sample for each PhoB expression level. Repression of reporter activity was not observed at high PhoB expression levels.

Figure S5

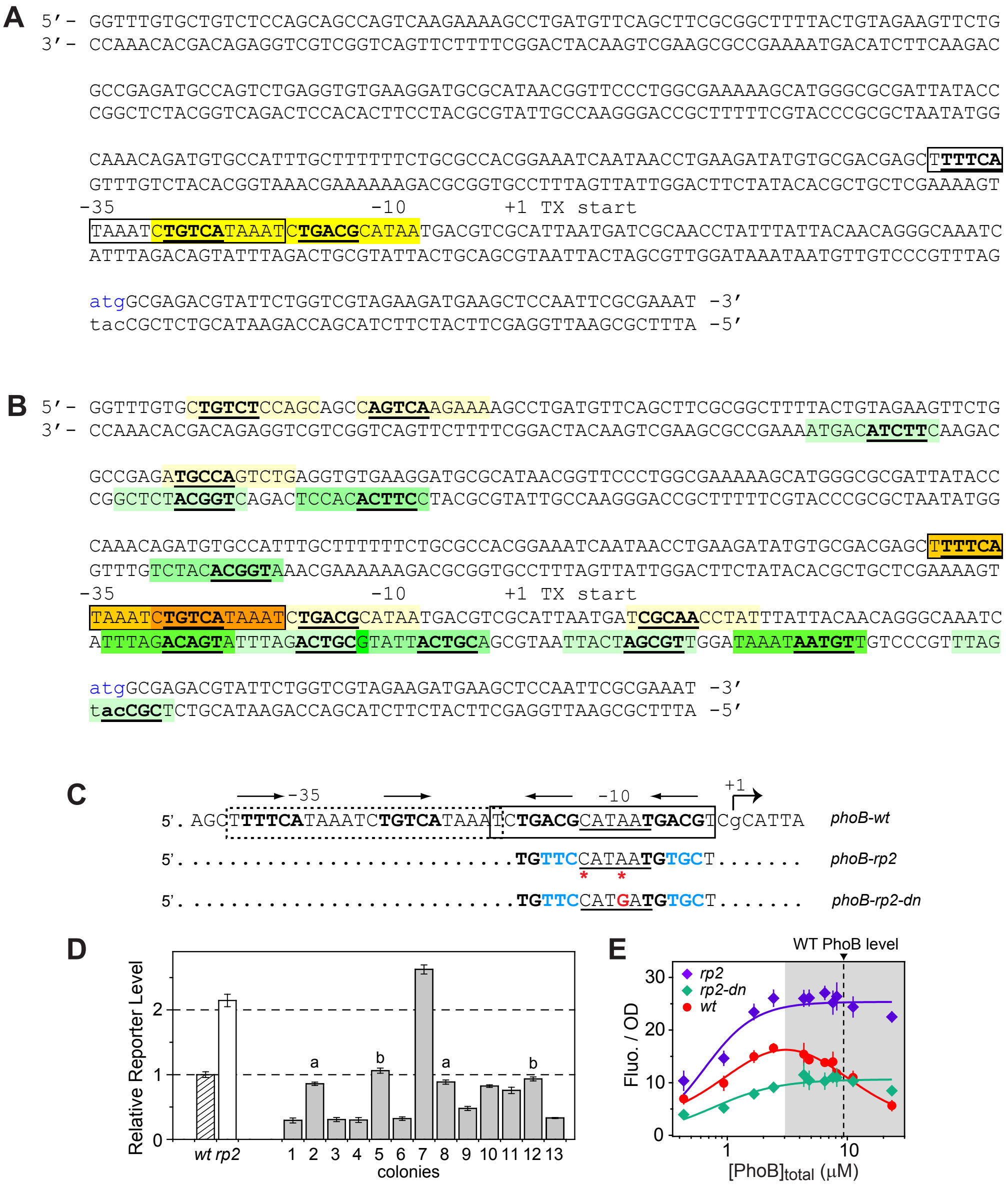

Figure S5. PhoB binding sites and screening of autoregulatory variants. Related to Figure 4 & 6. Full (A) and half (B) sites have been identified within the *phoB* promoter. A 22 bp full site besides the originally identified PHO box (boxed) is colored yellow in (A). Half sites on the sense strand and the antisense strand are shaded in yellow and green, respectively. Darker color indicates stronger sites. (C) Sequence of autoregulatory variants. The reporter plasmid pRG368 ($P_{phoB\text{-}rp2}$) contains mutations (blue letters) that disrupt the binding of PhoB~P to the repression site. The lack of repression leads to higher promoter activity than the WT reporter pJZG202. To screen for similar repression mutants with weak promoter strength and a comparable promoter output as the WT, two positions within the -10 region (labeled with red stars) were randomly mutated in pRG368 and the resulting YFP reporters were screened in strain BW25113. (D and E) Reporter activities of mutants. Several colonies displayed comparable reporter levels as the WT *phoB-yfp* reporter and four were sequenced (*a* and *b*). Two (*b*) have an identical sequence to WT while the other two (*a*) contain one single A to G mutation (red letter) in the -10 region. This variant was designated as *phoB-rp2-dn*. The resulting reporter plasmid pRG387 was placed in the non-autoregulatory strains and assayed for reporter output at different PhoB expression levels (E). Repression is not observed for *phoB-rp2-dn*. At the induced WT PhoB level (vertical dashed line) under Pi-depleted conditions, *phoB-rp2-dn* showed similar reporter output as the WT *phoB-yfp* reporter.

Figure S6

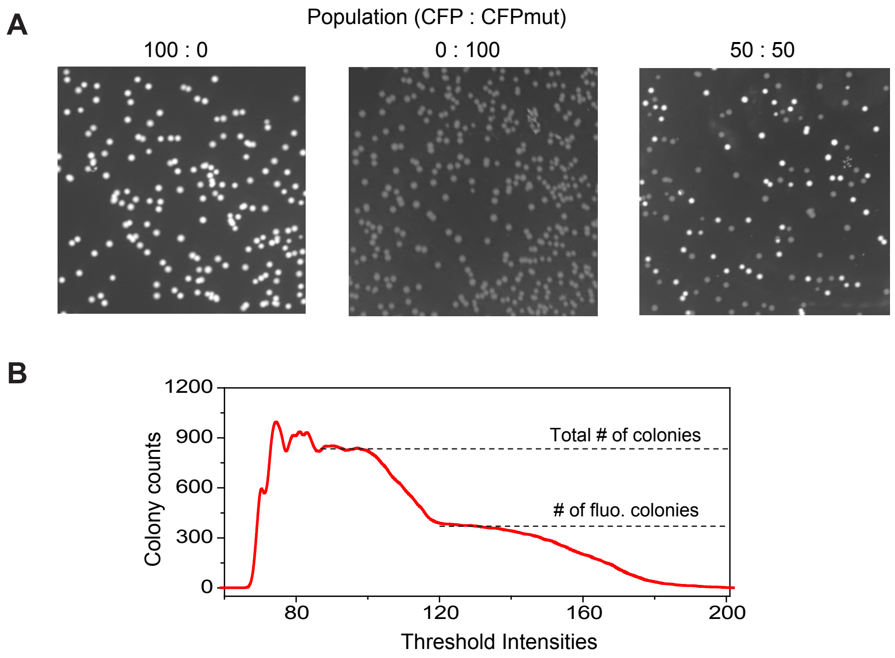

Figure S6. **Quantification of fluorescent colonies on LB plates. Related to Figure 6.**
(A) Fluorescent images of plates. Diluted cultures of RU1878 carrying either pRG411 (CFP) or pRG426 (CFPmut) were mixed in indicated proportions and spread on LB plates that contained chloramphenicol to ensure the maintenance of plasmids. Plate images were taken using a FluorChem Q imager with the following parameters: excitation 475/42 nm and emission 537/35 nm. Colonies with pRG411 showed strong fluorescence, while colonies with pRG426 displayed only minimal background fluorescence. (B) Quantification of colonies with an image thresholding algorithm. Fluorescent particles were counted with ImageJ with threshold values ranging from 1 to 255. The first plateau of colony counts occurs at lower threshold intensities, corresponding to all colonies, including both pRG411- and pRG426-carrying bacteria. The second plateau corresponds to bacterial colonies that carry pRG411 and display strong CFP fluorescence. The two plateaued colony numbers were used to calculate the population of fluorescent bacteria.

Table S1. Parameter values used in the model. Related to Figure 2 & 5.

Reaction / Parameter	Value	Source
Auto-phosphorylation		
k_k	0.06 s^{-1}	Fig. S1
k_{-k}	0.02 s^{-1}	Fig. S1
Phosphotransfer		
K_{dt}	0.9 μM	Fig. S1
k_1	0.15 $\mu M^{-1}\ s^{-1}$	Fig. S1
k_t	0.1 s^{-1}	Fig. S1
Dephosphorylation		
K_{dp}	0.9 μM	Fig. S1
k_2	0.15 $\mu M^{-1}\ s^{-1}$	Fig. S1
k_p	0.0072 s^{-1}	Fig. S1
RR auto-dephosphorylation		
k_y	$2.6\text{x}10^{-4}\ s^{-1}$	(Gao and Stock, 2013b)
DNA binding		
K_{DNA} *(phoB promoter)*	0.25 μM	Fig. S2
K_{DNA} *(phnC promoter)*	1.7 μM	Fig. S2
K_{RP}	4.9 μM	Fig. S2
Protein concentrations		
PhoB (Pi-replete)	0.45 μM	(Gao and Stock, 2013b; Gao and Stock, 2015)
PhoR (Pi-replete)	0.045 μM	(Gao and Stock, 2013b; Gao and Stock, 2015)
PhoB (Pi-depleted)	9.45 μM	(Gao and Stock, 2013b; Gao and Stock, 2015)
PhoR (Pi-depleted)	0.945 μM	(Gao and Stock, 2013b; Gao and Stock, 2015)
Protein dilution & degradation		
k_{dil}	$2\ \text{x}10^{-4}\ s^{-1}$	Estimated [a]
Protein production		
P_0	$9\ \text{x}10^{-5}\ s^{-1}$	Estimated [b]
P	*20* x P_0	Estimated [b]
P *(rp2)*	*41* x P_0	Estimated [b]

a. The rate is estimated from growth rates and PhoB stability (Gao et al., 2017; Gao and Stock, 2017).

b. Protein production rates are estimated based on the value of k_{dil} and protein concentrations to give respective steady-state PhoB levels.
